# Proteomic analysis of organic sulfur compound utilisation in *Advenella mimigardefordensis* strain DPN7^T^

**DOI:** 10.1371/journal.pone.0174256

**Published:** 2017-03-30

**Authors:** Christina Meinert, Ulrike Brandt, Viktoria Heine, Jessica Beyert, Sina Schmidl, Jan Hendrik Wübbeler, Birgit Voigt, Katharina Riedel, Alexander Steinbüchel

**Affiliations:** 1 Institut für Molekulare Mikrobiologie und Biotechnologie, Westfälische Wilhelms-Universität, Münster, Germany; 2 Institut für Mikrobiologie, Ernst-Moritz-Arndt-Universität, Greifswald, Germany; 3 Environmental Science Department, King Abdulaziz University, Jeddah, Saudi Arabia; Leibniz-Institut fur Naturstoff-Forschung und Infektionsbiologie eV Hans-Knoll-Institut, GERMANY

## Abstract

2-Mercaptosuccinate (MS) and 3,3´-ditiodipropionate (DTDP) were discussed as precursor substance for production of polythioesters (PTE). Therefore, degradation of MS and DTDP was investigated in *Advenella mimigardefordensis* strain DPN7^T^, applying differential proteomic analysis, gene deletion and enzyme assays. Protein extracts of cells cultivated with MS, DTDP or 3-sulfinopropionic acid (SP) were compared with those cultivated with propionate (P) and/or succinate (S). The chaperone DnaK (ratio DTDP/P 9.2, 3SP/P 4.0, MS/S 6.1, DTDP/S 6.2) and a Do-like serine protease (DegP) were increased during utilization of all organic sulfur compounds. Furthermore, a putative bacterioferritin (locus tag MIM_c12960) showed high abundance (ratio DTDP/P 5.3, 3SP/P 3.2, MS/S 4.8, DTDP/S 3.9) and is probably involved in a thiol-specific stress response. The deletion of two genes encoding transcriptional regulators (LysR (MIM_c31370) and Xre (MIM_c31360)) in the close proximity of the relevant genes of DTDP catabolism (*acdA*, *mdo* and the genes encoding the enzymes of the methylcitric acid cycle; *prpC*,*acnD*, *prpF* and *prpB*) showed that these two regulators are essential for growth of *A*. *mimigardefordensis* strain DPN7^T^ with DTDP and that they most probably regulate transcription of genes mandatory for this catabolic pathway. Furthermore, proteome analysis revealed a high abundance (ratio MS/S 10.9) of a hypothetical cupin-2-domain containing protein (MIM_c37420). This protein shows an amino acid sequence similarity of 60% to a newly identified MS dioxygenase from *Variovorax paradoxus* strain B4. Deletion of the gene and the adjacently located transcriptional regulator LysR, as well as heterologous expression of MIM_c37420, the putative mercaptosuccinate dioxygenase (Msdo) from *A*. *mimigardefordensis*, showed that this protein is the key enzyme of MS degradation in *A*. *mimigardefordensis* strain DPN7^T^ (*K*_*M*_ 0.2 mM, specific activity 17.1 μmol mg^-1^ min^-1^) and is controlled by LysR (MIM_c37410).

## Introduction

*Advenella mimigardefordensis* strain DPN7^T^ was first described by Wübbeler et al. in 2006 [1]. Initially, it was designated as *Tetrathiobacter mimigardefordensis* and in 2009 reclassified to the genus *Advenella*, which currently consists of five species [2–7]. Strains of this genus belong to the family *Alcaligenaceae* and have been detected in a variety of habitats [8]. They can perform diverse metabolic reactions, and some strains of this genus degrade xenobiotics [1,9].

*A*. *mimigardefordensis* strain DPN7^T^ was isolated because of its capability to utilize organic sulfur compounds such as the xenobiotic 3,3´-dithiodipropionic acid (DTDP), dibenzothiophene, taurine or 2-mercaptosuccinic acid (MS) as sole carbon source. Interestingly, *A*. *mimigardefordensis* strain DPN7^T^ is the only known strain known to metabolize DTDP as well as MS. This is interesting as both compounds were discussed as precursor substrates for polythioester (PTE) production [10].

PTEs represent an interesting class of biopolymers, whose constituents are linked by thioester bonds. Therefore, they comprise a sulfur-containing polymer backbone, which alters the thermophysical properties of the polymer, resulting in increased thermal stability and a higher degree of crystallinity in comparison to the structurally related polyhydroxyalkanoates [11,2]. PTEs could replace synthetic plastics derived from petrochemicals, especially if biologically persistent polymers are required. However, the production costs must be lowered significantly and the yield of the polymer must be increased [12]. The first chemical production of PTEs was described in 1951 by Marvel and Kotch [13]. In 2001, biotechnical production of PTEs has been reported by Lütke-Eversloh and colleagues [14] in strains of *Ralstonia eutropha* and *Escherichia coli*. In 2012, PTE homopolymer production starting from DTDP was successfully implemented [15] in *A*. *mimigardefordensis* strain DPN7^T^. The cellular content of the synthesized homopolymer amounted close to 25% (wt/wt, CDW). A polymer consisting of MS could not yet be produced, however, this compound could be interesting because of its potential crosslinking properties. Prior to polymerization, MS needs to be converted into the corresponding CoA-thioester and afterwards has to be accepted by a PHA synthase to achieve polymer production.

In comparison to other thiols, (e.g. 3-mercaptopropionic acid (3MP) an intermediate of DTDP catabolism [16]. MS exhibits some interesting characteristics, e.g. it acts as reducing agent due to the two carboxylic groups and as it is a thiol derivative, it can be used as a capping agent in nanoparticle production [17]. Furthermore, MS forms metal chelates with different metal ions [18,19], and it can act as a mono-, bi- or tridentate ligand since it possesses a thiol group as well as two carboxylic groups [20].

Microbial degradation of MS was first reported in 1968 by Hall and Berk [21]. They observed MS consumption by an isolate of the *Alcaligenaceae*. Since then only a few studies investigated the consumption of this compound. In 1993, MS was shown to be a substrate for organolithotrophic growth of *Rhodopseudomonas* sp. strain BB1. This strain first grows photoorganotrophically utilising fumarate, the MS degradation product after elimination of sulfide, and subsequently grows phototrophically using the latter [22]. Another study reported the isolation of *Advenellaincenata*, *Achromobacter xylosoxidans* and *Variovorax paradoxus* B4 based on their ability to utilize MS as the sole source of carbon and energy. The degradation pathway was then investigated in *V*. *paradoxus* B4 [10].

**Fig 1:**
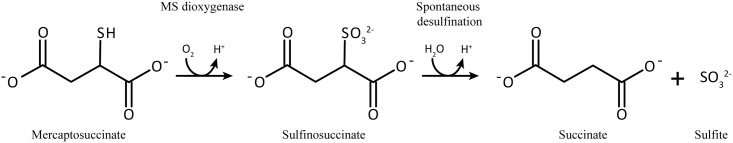
Degradation of 2-mercaptosuccinate in *Variovorax paradoxus* B4 [23].

After application of transposon mutagenesis, genomic and proteomic studies as well as enzymatic verification of the key reaction in MS degradation, a catabolic pathway was recently presented [23,24,25]. First, MS is oxidized by a newly identified enzyme referred to as mercaptosuccinate dioxygenase (Msdo) [23] forming sulfinosuccinate (Fig 1). The latter is spontaneously hydrolyzed to succinate and sulfite in aqueous solutions.

Detoxification of the sulfite in strain B4 is realized by a putative molybdopterin oxidoreductase or a rhodanese domain-containing protein, which requires a molybdenum cofactor [25]. Based on genomic data, possibilities for the detoxification of sulfite in *A*. *mimigardefordensis* strain DPN7^T^ were analyzed [3] and different pathways diverting from the detoxification system of *V*. *paradoxus* strain B4 were postulated. Thus, sulfite oxidation yielding sulfate is either achieved by sulfite oxidases (E.C.1.8.3.1) or disulfide dehydrogenases (E.C.1.8.2.1) [26,27]. The occurring sulfate is subsequently exported by a membrane protein belonging to the PSE (putative sulfate exporter) family encoded by a gene with the locus tag MIM_c23530 [3].

All attempts to obtain information regarding the degradation of MS in *A*. *mimigardefordensis* strain DPN7^T^ applying transposon mutagenesis using Tn*5*::*mob* were not successful, while this technique was very helpful to unravel the DTDP catabolism [1,16]. The import of DTDP into the cell is achieved via a hitherto unknown transport system. DTDP is then cleaved into two molecules of 3MP by the disulfide reductase LpdA (MIM_c19220). Afterwards, the 3MP dioxygenase (Mdo, MIM_c31400) incorporates two oxygen molecules into 3MP forming 3-sulfinopropionic acid (3SP). 3SP is then activated by the CoA-ligase SucCD (MIM_c18280/18290) yielding 3-sulfinopropionyl-CoA which is subsequently desulfurized by the desulfinase Acd (MIM_c31390). This reaction yields propionyl-CoA, which is finally catabolized via the methylcitric acid cycle [16, 28, 29,30]. The general adaption of the cell to the presence of DTDP, the toxic intermediate 3MP and MS is still unknown in this microorganism [1,6,15].

In 2014 the genome of *A*. *mimigardefordensis* strain DPN7^T^ was sequenced [3] and therefore “omics” strategies can now be pursued. We chose a proteomic approach to analyse changes in the protein profiles of cells of this strain during cultivation with the organic sulfur compounds MS and DTDP compared to its growth with succinate on succinate and propionate. As mentioned above, DTDP and MS were discussed as potential precursor substrates for production of PTEs. Therefore, the understanding of cellular adaptations to these organic sulfur compounds is of great interest. Moreover, it offers new perspectives in the enhancement of the polymer production in *A*. *mimigardefordensis* strain DPN7^T^. As described previously, the thiol 3MP occurs as an intermediate during degradation of DTDP. Hence it is also interesting to compare the stress response to the free thiols during utilization of the secondary thiol MS and 3MP in *A*. *mimigardefordensis* strain DPN7^T^.

## Materials and methods

### Growth conditions and cell harvest

All strains used in this study are listed in Table 1. Cells of *E*. *coli* were inoculated in Lysogeny Broth medium [31] at 37°C on a rotary shaker (New Brunswick Scientific Co., Inc., NJ, USA) at 130 rpm with addition of applicable antibiotics (ampicillin (Ap) 75 μg ml^-1^; tetracycline (Tc) 12.5 μg ml^-1^; chloramphenicol (Cm) 34 μg/ ml), if necessary.

**Table 1:** Strains and plasmids used in this study.

Strain or plasmid	Characteristics^a^	Source/Reference
Strains		
*Escherichia coli* Top 10	F^-^, *mcrA*, Δ(*mrr-hsdRMA-mcrBC*), *rpsL*, *nupG*, *80lacZ*Δ*M15*, Δ*lacX74*, *deoR*, *recA1*, *araD139*, Δ(*ara-leu*)*7697*, *galU*, *glaK*, *endA1*	Invitrogen
*E*. *coli* S17-1	*Thi-1*, *proA*, *hsdR17* (rK-mK+), *recA1*, *Tra*-genes of plasmid RP4 integrated into the genome	[32]
*E*. *coli* BL21(DE3) pLys	F^-^, *ompT*, *hsdS*_*B*_ (r_B_^-^,m_B_^-^), *gal*, *dcm* (DE3)/pLysS(Cm^r^)	Novagen
*A*. *mimigardefordensis* strain DPN7^T^	Type strain, DTDP and MS degrading	[1]
*A*. *mimigardefordensis* Δ*lysR* (MIM_c37410)	No growth with MS	This study
*A*. *mimigardefordensis* Δ*msdo* (MIM_c37420)	No growth with MS	This study
*A*. *mimigardefordensis* ΔMimc_14530	Wildtype phenotype	This study
*A*. *mimigardefordensis* ΔMimc_37450–37480	Wildtype phenotype	This study
*A*. *mimigardefordensis* Δ*lysR*Δ*xre* (Mim_c31370,MIM_c31360)	No growth with DTDP and propionate	This study
*A*. *mimigardefordensis* Δ*xre*Δ*prpC* (MIM_c31360,Mim_c31350)	No growth with DTDP	This study
Plasmids		
pJET1.2/blunt	*Bla*, *rep(pMB1)*, *eco47IR*	ThermoFisher Scientific
pJQ200mp18Tc	Tc^R^, suicide vector for gene deletion	[33]
pET-19b	pBR22 *ori*, Ap^r^, T7*lac*, His_6_-N-terminal tag	Novagen

^a^Abbreviations for antibiotic resistance genes and genotypes of *E*. *coli* according to [34].

Cells of *A*. *mimigardefordensis* strain DPN7^T^ were cultivated with nutrient broth or mineral salt medium (MSM) [35], which was supplemented with either 60 mM succinate, MS or DTDP, at 30°C. Liquid cultures were incubated on a rotary shaker in Erlenmeyer flasks without baffles. Growth was monitored via a Klett-Summerson photometer (Manostat Corporation, NY, USA) or an Ultrospec 2000 photometer (Pharmacia Biotech, Uppsala, Sweden). Precultures were incubated for 20 h, and main cultures of 400 ml for growth experiments and proteomic studies were inoculated to 30–40 KU at t = 0 h. For proteome analysis cells were harvested 6 h after the cultures had entered stationary phase by centrifugation for 30 min at 4°C and 7,690 x *g* (Hettich Universal 320 R, Andreas Hettich GmbH & Co KG, Tuttlingen, Germany). Cell pellets were stored at -20°C if not used immediately, whereas the supernatants were discarded. Solid media contained 20 mM carbon source and 1.8% (wt/vol) Bacto-Agar^™^ (Becton, Dickinson and Company, New Jersey, USA).

### Preparation of protein samples for proteome analysis

Cell pellets of cultures were suspended in 30 ml buffer (8 M urea, 2% (vol/vol) Triton X-114 and 2% (wt/vol) sodium dodecylsulfate (SDS)). The volume was adjusted with H_2_O_bidest_ to a total of 50 ml. Cell disruption was achieved using a French-Press^®^ (French^®^Pressure Cells and Press, Amicon, MD, USA) employing six to seven passages at 1,000 MPa per sample. Afterwards, samples were centrifuged at 60,000 x *g* and 4°C (Sorvall Discovery^™^90SE, Thermo Scientific, Fischer Scientific GmbH, Schwerte, Germany) to remove cell debris. 15 ml aliquots of the supernatant were transferred to 50 ml reaction tubes and stored at –20°C, if not used immediately. Protein extraction was achieved with phenol as described previously by Raberg et al.[36], differing only in the use of approx. 7.5 ml supernatant treated with 15 ml of Tris-saturated phenol. The protein pellet was air dried and stored at -20°C.

### Isoelectric focusing (1^st^ dimension) for 2D-PAGE

Prior to isoelectric focusing (IEF) the protein pellet was rehydrated in an appropriate amount (0.75–2.0 ml) of rehydration buffer (9 M urea, 4% (wt/vol) 3-[(3-cholamidopropyl)dimethylammonio]-1-propanesulfonate (CHAPS), 100 mM dithiothreitol (DTT)) overnight at 4°C. Samples were centrifuged at 7,300 x g and 4°C for 15 min to remove unsolved protein residues. Supernatants were transferred to 1.5 ml reaction tubes and stored at 4°C. A total amount of 1.5 mg protein was loaded onto IPG strips (ReadyStrip^™^ IPG strips, pH 5–8, 17 cm length, Biorad, Hercules, USA), and passive rehydration of the strips was performed overnight at room temperature. Prior to focusing, strips were transferred to the focusing tray and again overlaid with mineral oil. Thereafter, the following voltages were applied in the PROTEAN^®^ IEF cell (Biorad, USA): 250 V (250 Vh), 500 V (500 Vh), 1,000 V (1,000 Vh), 6,000 V (108,000 Vh), and 500 V (until further use).

### SDS-PAGE (2^nd^ dimension) for 2D-PAGE

The IPG strips were prepared and equilibrated for the 12.5% (wt/vol) polyacrylamide gels (200 mm x 200 mm x 1 mm) as described previously [23]. To this end, strips were fixed onto the gels with sealing solution (1% agarose, wt/vol, spatula-tip of bromophenol blue) and the SDS-polyacrylamide gel electrophoresis (PAGE) was performed in a DODECA Cell (PROTEAN^®^ plus DODECA^™^ Cell, Biorad, Hercules, USA) filled with buffer (25 mM Tris/HCl, 192 mM glycine, 0.1% (wt/vol) SDS). PAGE was carried out at 15°C, and 5 V per gel were applied for 1.5 h. Afterwards, 20 V per gel were used until the dye front reached the end of the gels. The gels were stained for approx. 16 h with staining solution containing 0.4% Serva Blue G 250 (wt/vol), 45% methanol, and 9% acetic acid. Destaining was performed with a solution composed of 33% (vol/vol) methanol and 10% (vol/vol) acetic acid for 10 h and afterwards, the gels were stored in 10% acetic acid.

### Software-based analysis of 2D-gel images

After appropriate destaining, gels were scanned (Epson perfection V700 photo, Suwa, Japan) and analysed with Delta2D 4.2 software (Decodon GmbH, Greifswald, Germany) according to the manufacturer’s recommended practices and as described previously [37]. Four replicates of each treatment (different carbon sources) were generated and used for analysis. The warping was done manually, and the automatically identified spots were manually corrected. Quantities of the spots were calculated as volume percentage of the associated spot in relation to the total level of protein in the corresponding gel. Statistics were performed applying the analysis of variance (ANOVA) tool of Delta2D and the p-value was set at 0.05. Therefore, normalization of the spot volumes was performed automatically in Delta2D. The total spot quantity was set to 100% on each gel image and the proportion of each spot was calculated. Spots, which possessed a higher standard deviation than 30%, were excluded from further analysis. A ratio was formed based on the mean spot volume % of each group of replicates. Spots with significant differences in their spot volume regarding the different treatments were identified applying a negative filter in the ranges 0.5–2.0. Spots with a more than two-fold increased spot volume and present on gels derived from cultures grown with DTDP or MS were analysed with matrix-assisted laser desorption/ionization-time-of-flight-tandem mass spectrometry (MALDI-TOF-MS/MS) analysis.

### Mass spectrometry

Proteins were cut from the SDS-Gels using a spot picker of 1.5 mm diameter (Biostep, Jahnsdorf, Germany) and transferred to 1.5 ml reaction tubes containing 20 μl 10% (v/v) acetic acid until used for mass spectrometry. Protein samples were prepared for MALDI-TOF-MS/MS as described previously [38]. MALDI-TOF-MS/MS analysis was performed using a 4800 Proteomics Analyzer (AB Sciex, Framingham, MA, USA). The spectra were recorded with a focus mass of 2000 Da in a reflector mode in a mass range from 900 to 3700 Da. Calibration of mass spectrometry data was performed as described in detail by Wolf et al [38]. Peak lists from MS and MS/MS were searched against the proteome database of *A*. *mimigardefordensis* strain DPN7^T^ using the Mascot engine (version 2.1.0.4).

### Generation of deletion mutants

Deletion of target genes was performed applying the suicide plasmid pJQ200mp18Tc [33] containing the ligated flanking regions adjacent to the target gene. After transfer of the construct to *A*. *mimigardefordensis* strain DPN7^T^ by conjugation with *E*. *coli* S17-1 as donor, the target genes were deleted by homologous recombination with the flanking regions within the plasmid.

### Isolation and transfer of DNA

Chromosomal DNA of *A*. *mimigardefordensis* strain DPN7^T^ was isolated according to Marmur [39]. Plasmid DNA was isolated from cells using the GeneJET plasmid miniprep kit from Fermentas (St. Leon-Rot, Germany) following the manufacturer´s instructions. Isolation of DNA fragments was realized using the peqGOLD gel extraction kit (PEQlab Biotechnology GmbH, Erlangen, Germany). Competent cells of *E*. *coli* were prepared and transformed by the CaCl_2_ method [31]. Plasmid DNA from *E*. *coli* was delivered to cells of *A*. *mimigardefordensis* strain DPN7^T^ by conjugation [40].

### Modification of DNA

PCR reactions for amplification of DNA were performed using the Phusion High-Fidelity Polymerase (Fermentas, St. Leon-Rot, Germany) and the Omnigene HBTR3CM DNA cycler (Hybaid, Heidelberg, Germany). Digestion of DNA was accomplished by use of restriction endonucleases (Fermentas, St. Leon-Rot, Germany). Ligation of restricted DNA fragments was performed using T4-Ligase (Invitrogen, Karlsruhe, Germany). Oligonucleotides were synthesized by MWG-Biotech (Ebersberg, Germany) and are listed in S1 Table.

### DNA sequencing

DNA sequencing reactions were set up in accordance with the requirements of MWG-Biotech (Ebersberg, Germany) and analysed by them. Verification of nucleotide sequences was carried out using the Seqman program (DNASTAR, Wisconsin, USA).

### Construction of gene deletion suicide plasmids

For successful deletion of target genes, flanking regions up- and downstream of the genes were amplified employing the oligonucleotides listed in S1 Table. The obtained flanks were ligated using T4-DNA ligase. Ligation products were amplified using the forward primer of the upstream located flank and the reverse primer of the downstream located flank of the gene of interest, respectively. Resulting PCR products were cloned into the *Xba*I site of pJQ200mp18Tc [33] to yield the corresponding suicide plasmids.

### Gene deletion using the *sacB* system

Transfer of the suicide plasmids was done by conjugation from the plasmid harbouring donor strain *E*. *coli* S17-1 to *A*. *mimigardefordensis* strain DPN7^T^ using the spot mating technique [33,40]. Mutants were identified on nutrient broth agar plates containing 15% (wt/vol) saccharose and on mineral salt medium agar plates containing 12.5 μg ml^-1^ tetracycline and succinate as carbon source. Gene deletions were verified by PCR analysis employing primers binding inside the gene of interest and outside of the flanking regions (S1 Table) and sequencing.

### Cloning, expression, and purification of Mercaptosuccinate dioxygenase (Msdo) of *A*. *mimigardefordensis* strain DPN7^T^

Amplification of *msdo* (MIM_c37420) was carried out with Phusion High-Fidelity DNA Polymerase (Thermo Scientific, Schwerte, Germany) using genomic DNA of *A*. *mimigardefordensis* strain DPN7^T^ and primers listed in S1 Table. The obtained fragment was then cloned into the *Nde*I and *Xho*I sites of plasmid pET-19b. Cells of CaCl_2_-competent *E*. *coli* Top 10 were then transformed with the obtained plasmid [31]. The correctness of the insert was verified by sequencing. CaCl_2_-competent cells of *E*. *coli* BL21(DE3) pLysS were transformed with the confirmed plasmid for expression of *msdo*_*Am*_. The main culture was cultivated in 50 ml LB medium supplemented with ampicillin and chloramphenicol and inoculated with 0.5 ml of a 20 ml overnight grown preculture. The main culture was grown at 30°C on a rotary shaker and synthesis of the protein was induced at OD_600_ 0.5 with isopropyl 1-thio-β-D-galactopyranoside (IPTG) with a final concentration of 400 μM. The culture was incubated during protein expression conditions at 25°C at 120 rpm on a rotary shaker for another 24 h. Cells were harvested using a Universal 320 R centrifuge (Andreas Hettich GmbH & Co. KG, Tuttlingen, Germany) at 7,690 x *g* for 15 min and at 4°C. Cells were resuspended in binding buffer (50 mM Tris/HCl, pH 7.4, 500 mM NaCl, 20 mM imidazole) and afterwards disrupted by sonication (30 Hz, 50% amplitude, 1 min/ml) (Sonoplus HD2200 MS72, Bandelin Electronic GmbH & Co. KG, Berlin, Germany). To remove cell debris, the crude extract was centrifuged for 15 min at 15,000 x *g* and 4°C (Centrifuge 5424R, Eppendorf Hamburg, Germany). Msdo_*Am*_ was purified from the obtained supernatant using nickel-nitriloacetate (Ni-NTA) affinity chromatography via a His SpinTrap column (GE Healthcare, Uppsala, Sweden) following the manufacturer´s instructions. Elution of Msdo_*Am*_ was performed by an elution buffer containing 500 mM imidazole (50 mM Tris/HCl, pH 7.4, 500 mM NaCl, 500 mM imidazole). The purified enzyme was directly used for enzyme assays or stored on ice until further use. Protein concentrations were determined using a Bradford assay [41].

### Quantitative activity assay with Msdo_*Am*_

Activity measurements were performed using the oxygen sensor OXMR connected to picoammeter PA2000 and the software MicOx (Unisense, Aarhus, Denmark) according to the manufacturer´s instructions. To calibrate the oxygen electrode, the probe was incubated in a solution containing 0.1 M ascorbic acid and 0.1 M NaOH until the value for these anoxic conditions was stable. Afterwards the electrode was incubated in buffer thoroughly aerated with compressed air until a stable value was observed to obtain the threshold for oxygen-saturated conditions. Determinations of kinetic data were done in 100 mM Tris/HCl buffer (pH 7.4) with different concentrations of MS (0.05; 0.1; 0.5; 0.75; 1.0; 2.0; 5.0; 7.5; 10; 20; 30; 50; and 75 mM). The reaction was started by addition of 2 μg/ml of enzyme. Oxygen consumption other than catalyzed by the enzyme was ruled out by control reactions without enzyme or with denatured Msdo. All measurements were carried out in triplicate. Nonlinear regression was performed using the Solver add-in of Microsoft Office Excel 2010.

## Results and discussion

### Cultivation of *A*. *mimigardefordensis* strain DPN7^T^ for proteome analyses

**Fig 2:**
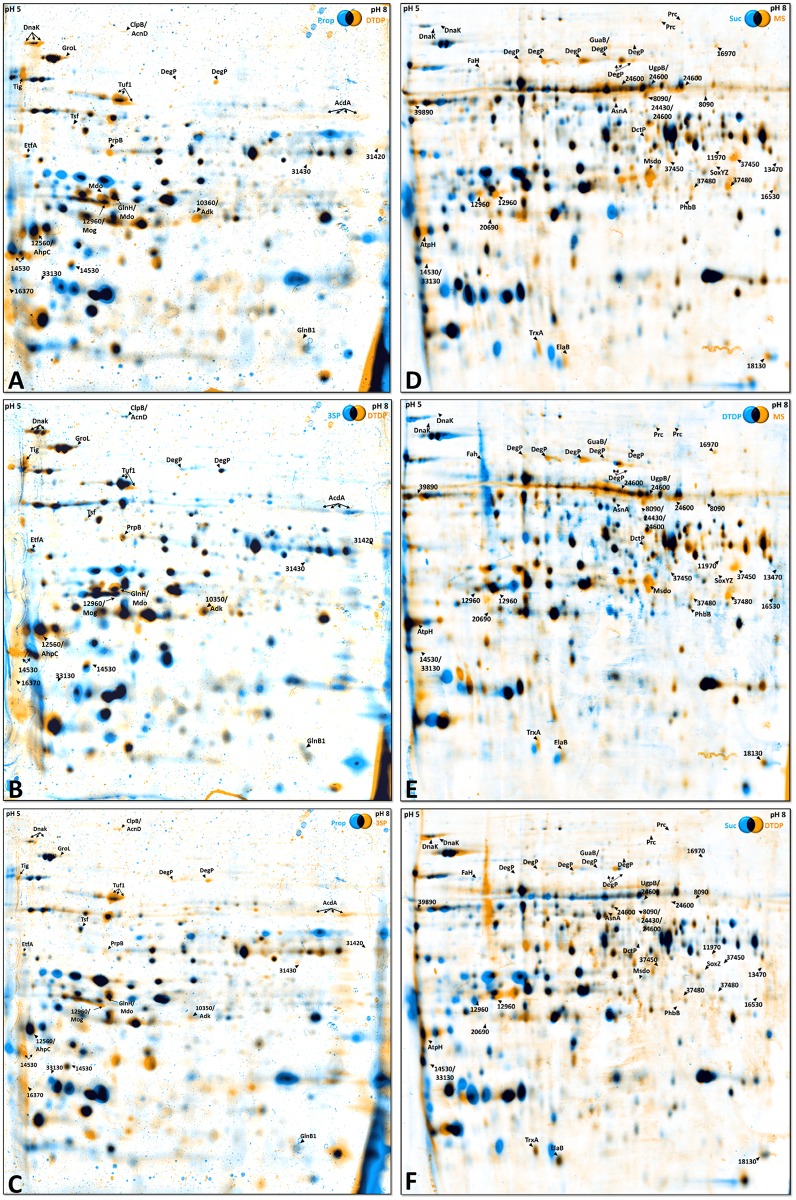
Variations of the proteome of *A*. *mimigardefordensis* strain DPN7^T^ cells cultivated with 3,3´-Dithiodipropionate (DTDP), Propionate (P) and 3-Sulfinopropionate (3SP) (A,B,C) and Mercaptosuccinate (MS), Succinate (S) and DTDP (D,E,F) displayed as dual channel views generated with Delta2D 4.2 software. Black spots represent protein spots with an equal amount of protein in both gels. Orange and blue spots represent proteins of the different substrate conditions as indicated on the gels. Spots marked with arrows comprise proteins with a significantly (≥ 3 fold) increased quantity during growth with: (A) DTDP in comparison to P, (B) 3SP in comparison to DTDP, (C) 3SP in comparison to P, (D) MS in comparison to S, (E) MS in comparison to DTDP and (F) DTDP in comparson to S. Cells from both cultures were harvested 6 hours after entering the stationary phase. 1.5 mg protein was loaded on IPG strips ranging from pH 5 to pH 8 and subsequently separated in the 2D-PAGE.

Two experimental designs were used, in order to display the proteome during the catabolism of MS and DTDP. The first approach aimed at the investigation metabolic processes during DTDP catabolism. For this, cells were cultivated in MSM with (i) propionate (P), the final degradation product of DTDP catabolism, (ii) 3SP, an intermediate of DTDP catabolism, or (iii) DTDP (Fig 2A, 2B and 2C). Differences in the proteomes regarding central metabolic processes should be minimized by choice of these carbon sources.

**Fig 3:**
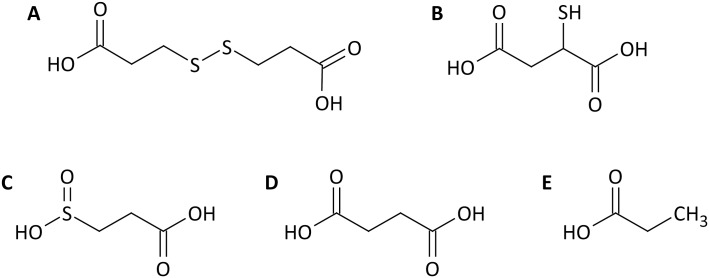
Chemical structures of the substrates used for cell cultivation prior to proteome analysis: A) 3,3´-dithiodipropionic acid, B) 2-mercaptosuccinc acid, C) 3-sulfinopropionic acid, D) succinic acid, E) propionic acid.

For the second approach, the proteome based analysis of MS degradation, cells of *A*. *mimigardefordensis* strain DPN7^T^ were either grown in MSM with (i) MS, (ii) DTDP or (iii) succinate (S). DTDP was used to identifiy upregulated proteins in the presence of both investigated organic sulfur compounds (Fig 2D, 2E and 2F) and succinate is the putative degradation product of MS. All carbon sources (Fig 3) were provided at a concentration of 60 mM.

Cells reached a higher cell density of approximately 480 Klett-Units (KU) with succinate and propionate, while only approximately 280 KU were reached when MS was supplied or only 350 KU were obtained when 3SP or DTDP were used. In addition, the doubling times (t_d_) of the cultures were higher when the organic sulfur compounds MS (t_d_ 15.75 h), DTDP (t_d_ 11.70 h) and 3SP (t_d_ 7.23 h) were fed in comparison to cultures with succinate (t_d_ 6.8 h) or propionate (t_d_ 6 h). Cells from all cultures were harvested 6 hours after entering the stationary growth phase.

### Detection and identification of proteins upregulated during cultivation with DTDP or MS

Utilisation of DTDP by *A*. *mimigardefordensis* strain DPN7^T^ was investigated in the first proteome experiment. In total, 560 spots were identified in the gel images. 98 of them, which showed increased spot volumes on gels from cells cultivated with DTDP or 3SP, respectively, were subjected to MALDI-TOF-MS/MS. Due to similar isoelectric points and molecular weights of the proteins, more than one protein species was identified for certain spots. Otherwise, due to isoforms of proteins in the 2D gels, the same protein species was identified in several spots. Therefore, 70 different proteins were identified during this experiment. The functions of the proteins were assigned using the KEGG database (http://www.genome.jp./kegg/).

**Fig 4:**
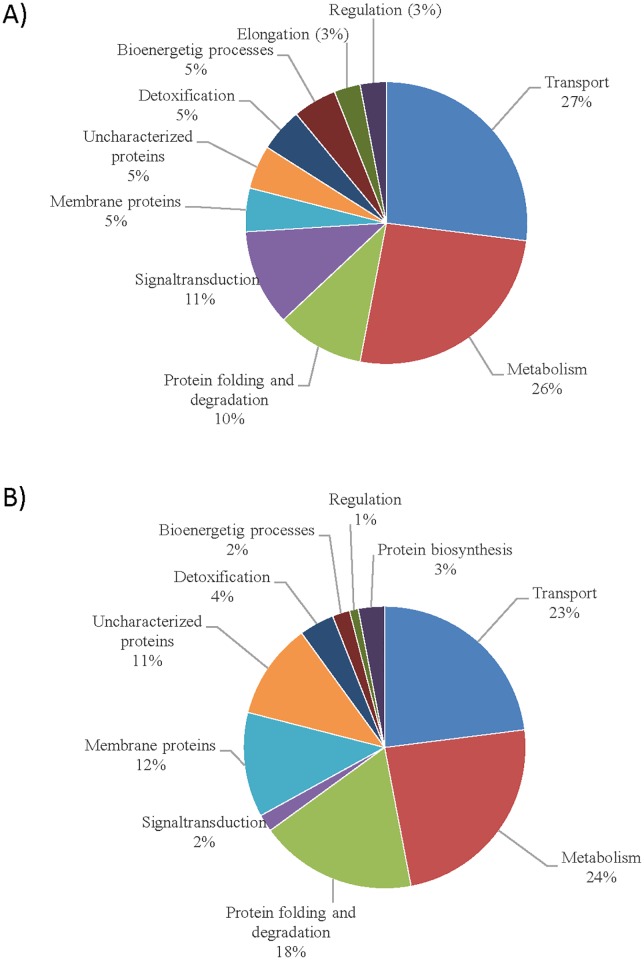
Functional categories of all differentially synthesized proteins from proteome analysis of cells cultivated with 3,3´-Dithiodipropionate (DTDP) in comparison to cells cultivated with propionate (A, in total 79 spots) and Mercaptosuccinate (MS) compared to succinate (B, in total 85 Spots). Protein functions were assigned using the KEGG (http://www.genome.jp./kegg/) database.

Accordingly, 27% of these proteins have functions in transport systems. Another 26% of all identified proteins perform metabolic reactions (Fig 4A). Proteins that are involved in protein folding, secretion or degradation, accounted to 9%, and proteins, which function in detoxification, make up another 5%. In total, 11% of all identified proteins were assigned to signal transduction. Furthermore, 5% were identified asmembrane associated proteins and another 5% act in bioenergetic processes. Elongation and regulation processes are represented by 3% each. The remaining 5% were annotated as uncharacterized proteins and could not be attributed to any function, yet.

This study focuses on proteins that showed an at least 3-fold higher quantity during cultivation with DTDP and MS in comparison to the corresponding controls, propionate (first experiment, regarding DTDP catabolism) and succinate (second experiment, regarding MS catabolism), respectively. An overview of the identified proteins is presented in Table 2.

**Table 2:** Proteins with an increased quantity (ratio ≥ 3) in cells of *A*. *mimigardefordensis* strain DPN7^T^ cultivated with 3,3´-Dithiodipropionate (DTDP) in comparison to cells cultivated with Propionate (P) or 3-Sulfinopropionate (3SP). A black-white color scale depicts the ratios of the different replicate groups (black- high, white- low).

Spot- Nr.	Locus tag (MIM_cXXXXX)	Protein	Gene	Ratio		
				DTDP/ P	3SP/ P	DTDP/ 3SP
49	09310	Glutamine-binding periplasmic protein	*glnH1*	**6.7**	**1.3**	**5.1**
61	10360	Putative phasin		**6.4**	**1.3**	**4.7**
3	11560	Chaperone protein DnaK	*dnaK*	**9.2**	**4.0**	**2.4**
4				**6.4**	**3.4**	**1.9**
127				**4.2**	**2.5**	**n.d.**
160	12560	Peptidyl-prolyl cis-trans isomerase	-	**3.7**	**2.0**	**n.d.**
152	12960	Ferritin-like domain-containing protein		**5.3**	**3.2**	**n.d.**
81	14530	Cupin 2 domain-containing protein		**4.0**	**4.4**	**0.8**
78				**5.6**	**4.3**	**1.2**
76				**3.6**	**1.4**	**2.6**
61	15800	Adenylate kinase	*adk*	**6.4**	**1.3**	**4.7**
60	15840	Superoxide dismutase [Fe]	*sodB*	**3.0**	**1.9**	**1.5**
62				**3.0**	**2.1**	**1.4**
167	16370	Putative peroxiredoxin-like protein		**3.6**	**1.3**	**n.d.**
180	16520	Electron transfer flavoprotein subunit alpha	*etfA*	**3.0**	**3.4**	**n.d.**
195	19340	Elongation factor Ts	*tsf*	**3.3**	**3.9**	**n.d.**
174	21350	Nitrogen regulatory protein P-II	*glnB1*	**3.1**	**2.2**	**n.d.**
130	22030	Trigger factor	*tig*	**4.2**	**2.1**	**n.d.**
2	22270	Chaperone protein ClpB	*clpB*	**5.2**	**7.4**	**0.7**
9	23840	Serine protease Do	*degP*	**8.5**	**7.5**	**1.1**
108				**4.2**	**12.0**	**0.3**
160	24360	Alkyl hydroperoxide reductase subunit C (EC 1.11.1.15)	*ahpC*	**3.7**	**2.0**	**n.d.**
152	27740	Molybdopterin adenylyltransferase	*mog*	**5.3**	**3.2**	**n.d.**
114	31320	Methylisocitrate lyase	*prpB*	**4.3**	**1.2**	**3.7**
2	31340	Fe/S-dependent 2-methylisocitrate dehydratase	*acnD*	**5.2**	**7.4**	**0.5**
13	31390	Acyl-CoA dehydrogenase	*acd*	**3.5**	**5.0**	**0.7**
14				**17.3**	**21.0**	**0.9**
16				**4.0**	**6.3**	**0.6**
17				**7.1**	**9.4**	**0.7**
138				**9.7**	**12.7**	**n.d.**
49	31400	3-Mercaptopropionate dioxygenase	*mdo*	**6.7**	**1.3**	**5.1**
152				**5.3**	**3.2**	**n.d.**
141	31420	Putative TctC extracytoplasmic solute-binding receptor, TTT family		**3.5**	**2.2**	**n.d.**
36	31430	Putative TctC extracytoplasmic solute-binding receptor, TTT family		**3.3**	**3.1**	**0.9**
5	32020	60 kDa Chaperonin GroEL	*groL*	**3.9**	**3.0**	**1.3**
90	33130	Putative LysM domain-containing BON superfamily protein		**4.7**	**6.4**	**0.6**
115	40710	Elongation factor Tu	*tuf1*	**4.4**	**8.7**	**0.5**
188				**3.7**	**5.7**	**n.d.**
190				**6.1**	**5.3**	**n.d.**

During the second experiment, investigation of MS degradation, 364 protein spots were detected in the 2D gels. Samples from cultures with MS in comparison to cultures with succinate revealed a significantly increased spot volume (≥ 2 fold) for 156 protein spots after application of statistical procedures. From them, 130 proteins were subjected to MALDI-TOF-MS/MS analysis, in which, due to isoforms, 82 different protein species emerged.

Thereof, 25% of all identified proteins participate probably in metabolic processes (Fig 4B). Since most transmembrane proteins were lost during the protein isolation process, mostly periplasmic components of various transport systems were identified and represent 22% of the identified proteins. Another 18% of the proteins were assigned to protein folding and degradation; further 12% have been categorized as membrane-associated proteins. Uncharacterized proteins represent 11% of the spots. The remaining 12% are distributed to detoxification (4%), protein synthesis (3%), signal transduction (2%), bioenergetic processes (2%), and regulation (1%).

A list of all identified protein species including spot-number, locus-tag, and ratios based on the average spot volumes of the two groups is included in the supplemental material (S2 Table). In this part of the study, as mentioned above, we will focus on protein species that exhibited an increased quantity (≥ 3 fold) in cultures cultivated with MS in comparison to cultures cultivated with succinate or DTDP (Table 3).

**Table 3:** Proteins with an increased quantity (ratio ≥ 3) in cells of *A*. *mimigardefordensis* strain DPN7^T^ cultivated with Mercaptosuccinate (MS) in comparison to cells cultivated with 3,3´-Dithiodipropionate (DTDP) or Succinate (S). A black-white color scale depicts the ratios of the different replicate groups (black- high, white- low).

Spot- Nr.	Locus tag (MIM_cXXXXX)	Protein	Gene	Ratio		
				MS/ S	MS/ DTDP	DTDP/ S
80	01930	C4-dicarboxylate-binding periplasmic protein	*dctP*	**8.1**	**3.5**	**2.4**
131	07490	ATP synthase subunit delta	*atpH*	**4.1**	**0.8**	**5.2**
60	08090	Outer membrane porin protein		**6.7**	**4.4**	**1.5**
59				**4.0**	**1.2**	**3.3**
137	11560	Chaperone protein DnaK	*dnaK*	**6.1**	**n.v**	**6.2**
138				**3.3**	**0.8**	**4.5**
150	11970	Putative Bug-like extracytoplasmic solute-binding receptor, TTT family		**7.3**	**0.9**	**8.2**
29	12960	Ferritin-like domain-containing protein		**4.8**	**1.2**	**3.9**
15				**3.9**	**1.1**	**3.5**
151	13470	Putative Bug-like extracytoplasmic solute-binding receptor, TTT family		**4.1**	**0.5**	**8.6**
109	13540	Acetoacetyl-CoA reductase	*phbB*	**5.1**	**1.2**	**4.4**
128	14530	Mercaptosuccinic acid dioxygenase	*msdo*	**14.7**	**8.6**	**1.7**
157	16530	Putative protease, peptidase family M48		**4.0**	**1.1**	**3.8**
45	16690	Fumarylacetoacetase	*fah*	**3.3**	**1.7**	**2.0**
117	16970	Putative argininosuccinate lyase		**6.4**	**3.5**	**1.9**
126	18130	Hypothetical protein		**3.2**	**0.7**	**4.3**
119	18410	Thioredoxin	*trxA*	**3.4**	**2.4**	**1.4**
58	19070	Sn-glycerol-3-phosphate-binding periplasmic protein	*ugpB*	**4.8**	**3.7**	**1.3**
123	20690	Putative enoyl-CoA hydratase		**3.2**	**1.0**	**3.2**
40	21640	Inosine-5'-monophosphate dehydrogenase	*guaB*	**3.2**	**1.9**	**1.7**
33	22960	Putative tail-specific protease	*prc*	**7.7**	**0.9**	**8.8**
136				**5.8**	**1.0**	**5.9**
141	23840	Do-like serine protease	*degP*	**10.3**	**7.3**	**1.4**
142				**8.5**	**8.8**	**1.0**
42				**7.1**	**0.7**	**10.2**
145				**5.8**	**1.4**	**4.3**
43				**5.8**	**3.8**	**1.5**
144				**5.7**	**1.8**	**3.1**
38				**4.0**	**0.7**	**6.1**
40				**3.2**	**1.9**	**1.7**
60	24430	Putative CBS domain-containing nucleotidyltransferase		**6.7**	**4.4**	**1.5**
60	24600	Outer membrane porin protein		**6.7**	**4.4**	**1.5**
58				**4.8**	**3.7**	**1.3**
48				**4.1**	**2.0**	**2.0**
55				**3.1**	**2.9**	**1.0**
122	27900	Putative protein ElaB	*elaB*	**3.2**	**2.1**	**1.5**
61	28910	L-asparaginase	*ansA*	**3.4**	**2.2**	**1.6**
128	33130	Putative LysM domain-containing BON superfamily protein		**14.7**	**8.6**	**1.7**
91	36710	Sulphur oxidation protein	*soxYZ*	**7.6**	**1.7**	**4.5**
153	37420	Cupin 2 domain-containing protein		**10.9**	**n.v**	**1.4**
83	37450	Putative periplasmic amino acid-binding protein		**22.7**	**7.4**	**3.1**
85				**5.6**	**0.6**	**8.6**
102	37480	Amino acid ABC transporter ATP-binding protein		**7.0**	**2.3**	**3.1**
101				**6.8**	**7.5**	**0.9**
4	39890	Branched-chain amino acid ABC transporter substrate-binding protein		**3.7**	**1.6**	**2.3**

### Proteins with increased quantity in the presence of organic sulfur compounds

The organic sulfur compounds DTDP and MS were used for cell cultivation in both experiments. Since the catabolism of DTDP and MS varies, as described in this study, we expected different patterns in the proteome of cells cultivated with these compounds. Additionally, the organic sulfur compound 3SP was used in addition in the proteome experiment investigating the DTDP catabolism.

Proteins related to the stress response showed high quantities when DTDP, MS or 3SP was provided, e.g. DnaK (MIM_c11560) and a Do-like serine protease (MIM_c23840). In 2008, transposon-induced mutants revealed a link of DnaK and DnaJ (MIM_c11570) to the utilization of DTDP. Thereby, insertion of the transposon Tn*5*::*mob* in *dnaK* resulted in a DTDP-negative phenotype and insertion of Tn*5*::*mob* in *dnaJ* provoked a DTDP-leaky phenotype, respectively [16]. DnaK was identified in all gels of samples cultivated with one of the applied organic sulfur compounds (ratio DTDP/P up to 9.2, DTDP/S 6.2 and 4.5, 3SP/P 4.0 and 3.4 and MS/S 6.1 and 3.3).

Beside DnaK, the protease DegP (MS/S ratios from 3.2 up to 10.3; DTDP/S 1.0 up to 10.2; DTDP/P 8.4 and 4.2; 3SP/P 7.5 and 12.0) showed high protein amounts in the gels of samples cultivated with the organic sulfur compounds. Additionally, the trigger factor (TF, MIM_c22030, DTDP/P 4.23) and the heat shock proteins GroL (MIMc_32020, ratio DTDP/P 3.9; 3SP/P 7.4) and ClpB (MIMc_22270, ratio DTDP/P 5.2; 3SP/P 7.4) showed higher protein levels during cultivation with DTDP and 3SP. Interestingly, these proteins were not identified in the proteomic analyses of MS and DTDP degradation, when succinate served as control (S2 Table). Overproduction of TF can likewise inhibit cell division, which can also be achieved if a critical amount of misfolded chaperones is present [42]. This might be an explanation for the reduced cell density and prolonged *lag* phase of the cultures cultivated with the organic sulfur compounds. Most chaperones are formed constitutively and are upregulated when heat shock conditions or oxidative stress occur [43]. In *E*. *coli*, DegP is also synthesized under heat shock conditions and functions in protein degradation in the periplasm of the cells [44,45].

Beside the chaperones and proteases, peptidyl-prolyl-isomerase (MIM_c12560) was identified in protein spots with a significantly increased spot volume (ratio DTDP/P 2.4–3.7; 3SP/P 1.8–2.0) in cells cultivated with DTDP and 3SP. These proteins are involved in *in situ* repair mechanisms of damaged amino acid residues [46]. It is likely that this protein is also present and most probably upregulated when the cells use MS as the carbon source, even if this protein was not identified under these conditions.

These observations clearly show that the organic sulfur compounds, which were used as carbon sources, or their intermediates evoke a strong stress response in cells of *A*. *mimigardefordensis* strain DPN7^T^. MS itself is a thiol, and during degradation of DTDP the thiol 3MP occurs as an intermediate. Thiols in cells have important properties in active sites of enzymes or coenzymes and in the detoxification of chemicals, or they act intracellularly as reducing agents. In this context, the ratio of thiols and disulfides is crucial for the intracellular redox balance. If this ratio is significantly disturbed, for example, due to the presence of other thiols (e.g. 3MP or MS), reductive processes occur and Fe^3+^ is reduced to Fe^2+^. The reduced iron eventually is the cause of formation of hydroxide ions and hydroxyl radicals, which may cause DNA damage and evoke an oxidative stress response [47,48].

In both proteome analyses, a protein with an abundance ratio ≥ 3 in all organic sulfur compounds containing samples was annotated as ferritin-like domain containing protein (MIM_c12960). Sequence analysis showed that this protein is most likely a bacterioferritin. These proteins belong to a superfamily of iron oxidation, storage, and mineralization proteins [49] and detoxify iron through oxidization of Fe^2+^ to Fe^3+^. During cultivation in the presence of organic sulfur compounds, it is likely that the detected bacterioferritin is part of the thiol-specific stress response in *A*. *mimigardefordensis* strain DPN7^T^.

**Fig 5:**
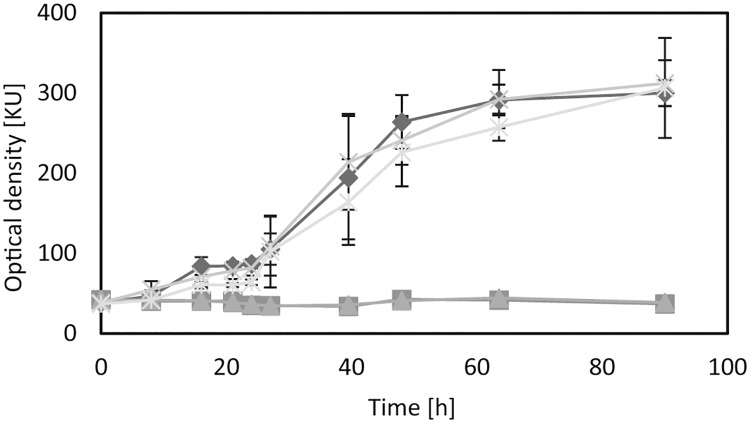
Utilization of Mercaptosuccinate (MS) by cells of *A*. *mimigardefordensis* strain DPN7^T^.: *A*. *mimigardefordensis* Δ*lysR* (MIM_c37410, squares), *A*. *mimigardefordensis* Δ*msdo* (MIM_c37420, triangles), *A*. *mimigardefordensis* Δ14530 (crosses), *A*. *mimigardefordensis* Δ37430–37480 (stars), *A*. *mimigardefordensis* strain DPN7^T^ (diamonds).

A second protein also possessing an oxidase function is a cupin domain-containing protein, encoded by the gene with the locus tag MIM_c14530. It was detected on the gels with a ratio ≥ 3 when the proteomes of cells cultivated with DTDP, MS or 3SP were compared to the proteomes of cells cultivated with either succinate or propionate. The respective gene was deleted, but growth experiments showed that the absence of this gene had no influence on the catabolism of either carbon source (Fig 5). *In silico* analysis revealed another cupin-2 domain-containing protein (25% identical amino acids of MIM_c14530 to MIM_c02690), which might compensate the function of the deleted gene.

The last identified protein with an at least four-fold higher expression is a putative lysin motif (LysM) domain-containing BON superfamily protein (MIM_c33130). The LysM motif in combination with a BON domain is putatively found in proteins associating with phospholipid membranes and was identified with two membrane pore-forming domains of secretin and ion channels [50,51]. Deletion of this gene was not possible and therefore, its function remains speculative and requires further investigations.

### DTDP catabolism and its regulation

The transport of DTDP is most probably performed by an active transport system that uses a periplasmic substrate-binding protein. One goal of this study was to identify proteins which might be involved in this hitherto unknown DTDP import [16,28]. Some periplasmic components of transport systems putatively involved in DTDP uptake were detected during the proteome anlalysis.

First, the substrate binding protein of a glutamine transport system was detected, which showed an 6.7-fold increased abundance in cells grown with DTDP in comparison to cells grown with propionate and a ratio DTDP/S of 3.3. In addition, *glnQ*2 (MIM_c09330), the ATP-binding protein GlnQ2 of the same ABC transport system was detected with a 2.1 fold higher abundance in cultures cultivated with DTDP in comparison to propionate (S3 Table).

Furthermore, two putative extracytoplasmic solute-binding receptors (MIM_c31420, ratio DTDP/P 3.5 and MIM_c31430,ratio DTDP/P 3.3) of the TTT family, as well as four more periplasmic components of amino acid specific ABC transporters (S3 Table) were identified with high levels in gels with proteins from cultures fed with DTDP in comparison to samples from cultures fed with propionate.

Most of the proteins involved in DTDP catabolism were identified during the proteome analysis of cells cultivated with DTDP in comparison to cells cultivated with propionate. Mdo (MIM_c31400), which catalyzes the conversion of the 3MP to 3SP [16], was identified in two spots with ratios of 5.3 and 6.7, respectively. 3SP is activated to 3SP-CoA by a succinyl-CoA ligase (SucCD, MIM_c18280-18290) homolog [28]. Desulfination of the molecule is performed by an acyl-CoA dehydrogenase (Acd, MIM_c31390) yielding propionyl-CoA [29]. Acd was identified in several spots with ratios ranging from 3.5 to 17.3 (DTDP/P). Acd was also detected in the comparison of the proteome of cells cultivated with 3SP and propionate with ratios 3SP/P ranging from 4.7 to 21.0.

**Fig 6:**
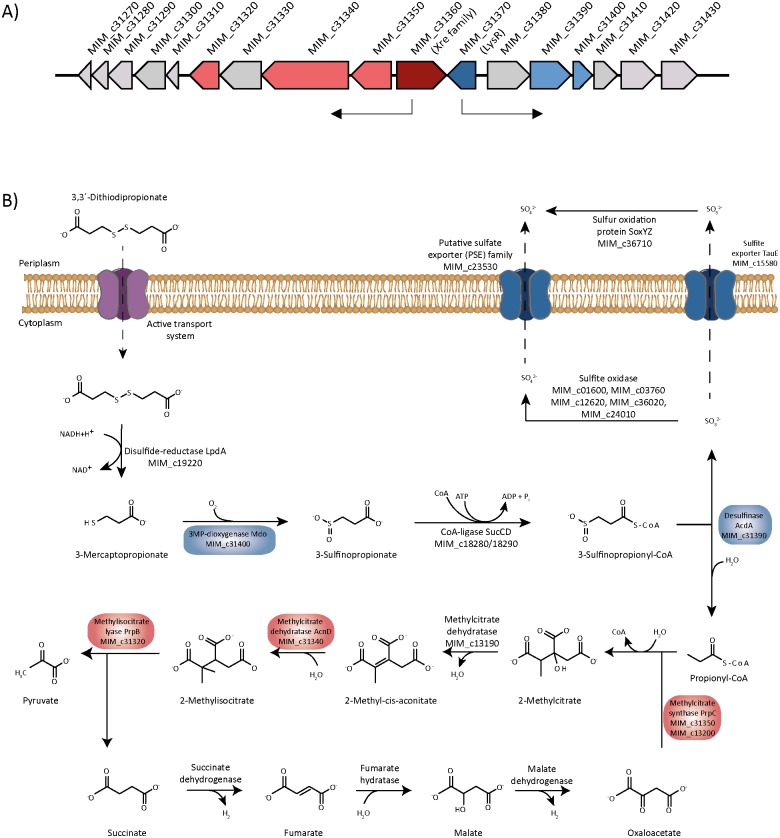
A) Gene cluster of DTDP catabolism in *A*. *mimigardefordensis* strain DPN7^T^ most probably regulated via XRE (red) and LysR (blue). Genes correspondig to the locus tags: MIM_c31270, putative membrane protein; MIM_c31280, putative membrane protein, DoxX family; MIM_c31290, hypothetical protein; MIM_c31300, hypothetical protein; MIM_c31310, hypothetical protein; MIM_c31320, methylisocitrate lyase; MIM_c31330, putative AcnD-accessory protein PrpF; MIM_c31340, Fe/S-dependent 2-methylisocitrate dehydratase; MIM_c31350, 2-methylcitrate synthase; **MIM_c31360, transcriptional regulator, XRE family**; **MIM_c31370, transcriptional regulator, LysR family**; MIM_c31380, acyl-CoA transferase, family III; MIM_c31390, desulfinase Acd; MIM_c31400, 3-mercaptopropionate dioxygenase Mdo; MIM_c31410, alkylhydroperoxidase AhpD core domain-containing protein; MIM_c31420, extracytoplasmic solute binding receptor TctC, TTT family; MIM_c31430, extracytoplasmic solute binding receptor, TctC, TTT family. B) Proteins under control of Xre and LysR which were identified during proteome analysis are indicated in red and blue, respectively.

**Fig 7:**
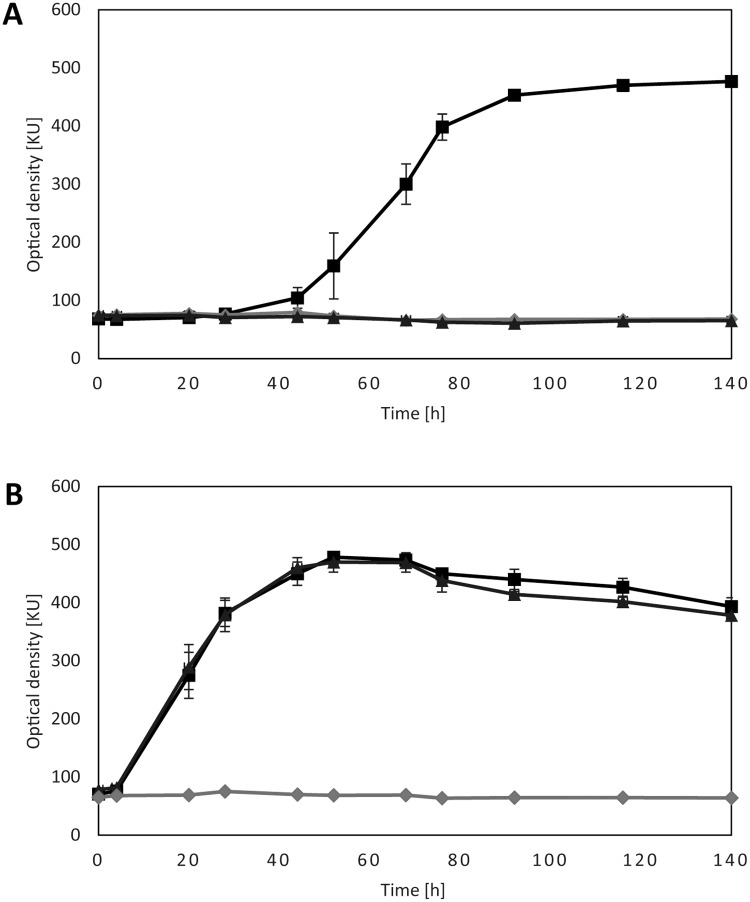
Growth of *A*. *mimigardefordensis* strain DPN7^T^, *A*. *mimigardefordensis* Δ*xre*Δl*ysR* and *A*. *mimigardefordensis* Δ*xre*Δ*prpC*, with 3,3´-ditiodipropionate (A) and propionate (B). *A*. *mimigardefordensis* strain DPN7^T^ (squares), *A*. *mimigardefordensis* Δ*xre*Δ*lysR* (triangles), *A*. *mimigardefordensis* Δ*xre*Δ*prpC* (diamonds). All experiments were carried out in triplicate, bars indicate the standard deviation.

A LysR transcriptional regulator (MIM_c31370) is located upstream of *mdo* and *acd* as shown in Fig 6A. The gene (MIM_c31370) was deleted in form of the double deletion mutant *A*. *mimigardefordensis* Δ*xre*Δ*lysR*, to investigate if this gene encodes the regulating element for the initial DTDP catabolism. *A*. *mimigardefordensis* Δ*xre*Δ*lysR* showed impaired growth with medium containing DTDP as sole carbon source (Fig 7A). LysR-type transcriptional regulators have been described as global transcriptional regulators that activate or repress single or operonic genes. Thereby, the regulator is predominantly located adjacent to the regulated genes and transcribed divergently[52], but it can also be located elsewhere in the bacterial chromosome [53,54]. Usually, LysR regulators need co-inducers which contribute to the feedback loop inducing or repressing the transcription of the corresponding genes. These molecules are mostly intermediates of the LysR regulated metabolic pathway [55,56,57]. The co-inducing element for the DTDP catabolism regulating LysR remains unknown.

Propionyl-CoA is metabolized via the methylcitric acid pathway in *A*. *mimigardefordensis* strain DPN7^T^, which is regulated by the xenobiotic response regulator (XRE, MIM_c31360), as described hereinafter. The regulator encoding gene is located upstream of the operon coding for genes of the methylcitric acid pathway. Two double deletion mutants *A*. *mimigardefordensis* Δ*lysR*Δ*xre* and *A*. *mimigardefordensis* Δ*xre*Δ*prpC* (MIM_c31350-31360) were generated in this study, and both double mutants lacked the ability to utilize DTDP as the sole source of carbon as shown in Fig 7A. In contrast to the propionate-negative phenotype of *A*. *mimigardefordensis* Δ*xre*Δ*prpC*, the compound could still be utilized by the deletion mutant Δ*lysR*Δ*prpC* (Fig 7B). *A*. *mimigardefordensis* strain DPN^7^ posesses a paralogue to prpC (MIM_c13200). Therefore, the DTDP-negative and especially propionate-negative phenotype results from the deletion of *xre*. The 2-methylisocitrate dehydratase (AcnD), the methylisocitrate lyase (PrpB) and the malate dehydrogenase (Mdh) were identified in the proteome analysis and were strongly formed with ratios of 5.2, 4.4 (Table 2) and 2.4 (S2 Table), respectively.

In addition to the genes involved in the degradation of DTDP, a putative phasin was identified with a 6.4-fold higher abundance during cultivation with DTDP than during cultivation with propionate. In comparison to 3SP, the protein showed a 4.7-fold increased expression during cultivation with DTDP. *A*. *mimigardefordensis* strain DPN7^T^ is able to synthesize PHAs and also a copolymer containing 3MP arising from DTDP degradation [1]. PHAs and their copolymers are accumulated in granula in the bacterial cells with phasins located on the granule surface [58]. Therefore, the formation of a phasin is not surprising.

### Utilization of MS

In total, 27 different protein species were particularly conspicuous in the proteomic analysis (Table 3) during MS utilization of *A*. *mimigardefordensis* strain DPN7^T^. Several identified proteins were associated with the stress response in *A*. *mimigardefordensis* strain DPN7^T^ during cultivation with the organic sulfur compound MS, including DnaK (ratios MS/S 6.1 and 3.3) and two putative proteases with the locus tags MIM_c16530 (ratio MS/S 4.0) and MIM_c22960, of which two isoforms were detected (ratios MS/S 7.7 and 5.8). Additionally, eight isoforms of the Do-like serine protease were spotted ranging in ratios from 3.2 to 10.3. Among the proteins, related to stress response are thioredoxin (ratio MS/S 3.4) and the sulfur oxidation protein (SoxYZ), whose spot volume was increased by a factor of 7.6.

Two of the conspicuous proteins represent porins (MIM_c08090 and MIM_c04600), and the six corresponding spots showed significantly increased spot volumes in the gels (MIM_c08090, ratio MS/S 6.7 and 4.0; MIM_c04600, ratio MS/S 6.7, 4.8, 4.1 and 3.1). Seven protein species were associated with several active transport systems, including the tripartite tricarboxylate transporter family, tripartite ATP-independent periplasmic transporters, and ABC-transport systems. Two proteins of an ABC-transporter were identified, in two spots each during proteome analysis with increased quantity in gels from samples cultivated with MS in comparison to cells cultivated with succinate. These proteins are a putative periplasmic amino acid-binding protein (MIM_c37450, ratio MS/S 22.7 and 5.6) and an ATP-binding protein of the putative amino acid transport system (MIM_c37480, ratio MS/S 7.0 and 6.8). Based on these data, an MS transport system could not be predicted. We assumed that the transporter with the two identified proteins may be involved in the uptake of MS into the cells, but deletion of the complete transport system (MIM_c37450-MIM_c37480) had no influence on the growth behaviour of *A*. *mimigardefordensis* with all tested carbon sources, including MS and DTDP (Fig 5).

Another protein (MIM_c37420) encoded in this gene neighborhood showed a 10.9-fold increased quantity during cultivation with MS. The gene product was annotated as a cupin 2 domain-containing protein and is one of two proteins of this type identified in this study. The other cupin 2 domain-containing protein (MIM_c14530, ratio MS/S 14.7) was described above in detail. The detailed discussion of the cupin 2 domain-containing protein with the locus tag MIM_c37420 follows in the section below.

### LysR regulates the gene region encoding Msdo_DPN7_, the key enzyme of MS degradation

As mentioned above, during proteome analysis two cupin 2 domain-containing proteins MIMc_14530 and MIMc_37420 showed high abundance in gels loaded with proteins from cultures cultivated with MS in comparison to cultures with succinate. We generated deletion mutants of both genes. Growth experiments with the mutants revealed that only the deletion of MIM_c37420 resulted in restricted growth of *A*. *mimigardefordensis* strain DPN7^T^ with MS as the sole carbon source (Fig 4).

Recently, a Msdo was identified and characterized in *V*. *paradoxus* strain B4. The enzyme was described as a non-heme iron metalloenzyme that is a structural analog of the well-characterized cysteine dioxygenase [23,24] comprising the typical cupin motifs of dioxygenases. MIM_c37420 showed an amino acid sequence identity of 60% to the Msdo_B4_, and the two cupin motifs were also identified (S4 Fig). Similar to *msdo*_B4_, MIM_c37420 from *A*. *mimigardefordensis* strain DPN7^T^ is clustered with genes coding for a rhodanese domain-containing protein and a cystathionine β-lyase, supporting the assumption of MIM_c37420 coding for a putative Msdo_DPN7_. In *V*. *paradoxus* B4, Msdo_B4_ catalyzes the oxidation of MS to sulfinosuccinate. The latter is a very unstable compound that spontaneously disintegrates into succinate and sulfite [23].

**Fig 8:**
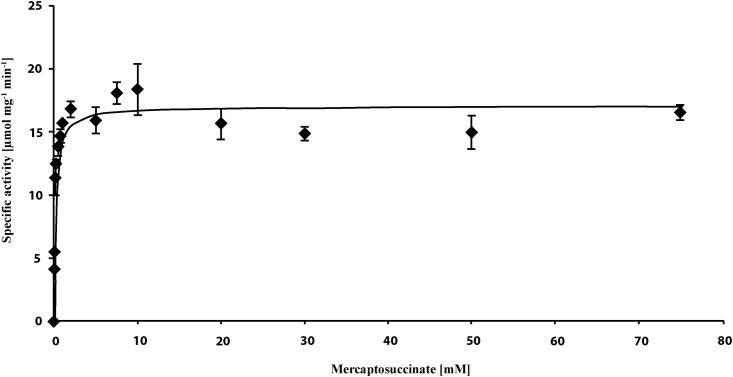
Kinetic data determined by measuring the oxygen consumption during conversion of MS to succinate by Msdo_DPN7._ The experiment was carried out in triplicate applying MS concentrations from 0.05 mM to 75 mM in 100 mM Tris/HCl buffer at pH 7.4.

In this study, overproduction of MIM_c37420, which is referred to as Msdo_DPN7_ in the following text, was accomplished with pET19b::*msdo*_DPN7_ in *E*. *coli* BL21 (DE3) pLysS (S3 Fig). Oxygen consumption of the enzymatic reaction was measured with different MS concentrations ranging from 0.05 to 75 mM (Fig 8). Based on these data, the apparent *K*_*M*_ value of 0.2 mM and a specific activity of 17.1 μmol mg^-1^ min^-1^ were determined, which is similar to the *K*_*M*_ value of 0.4 and the specific activity of 20 μmol mg^-1^ min^-1^ of Msdo_B4_ indicating that the Msdo_DPN7_ has a comparable affinity to the substrate.

**Fig 9:**
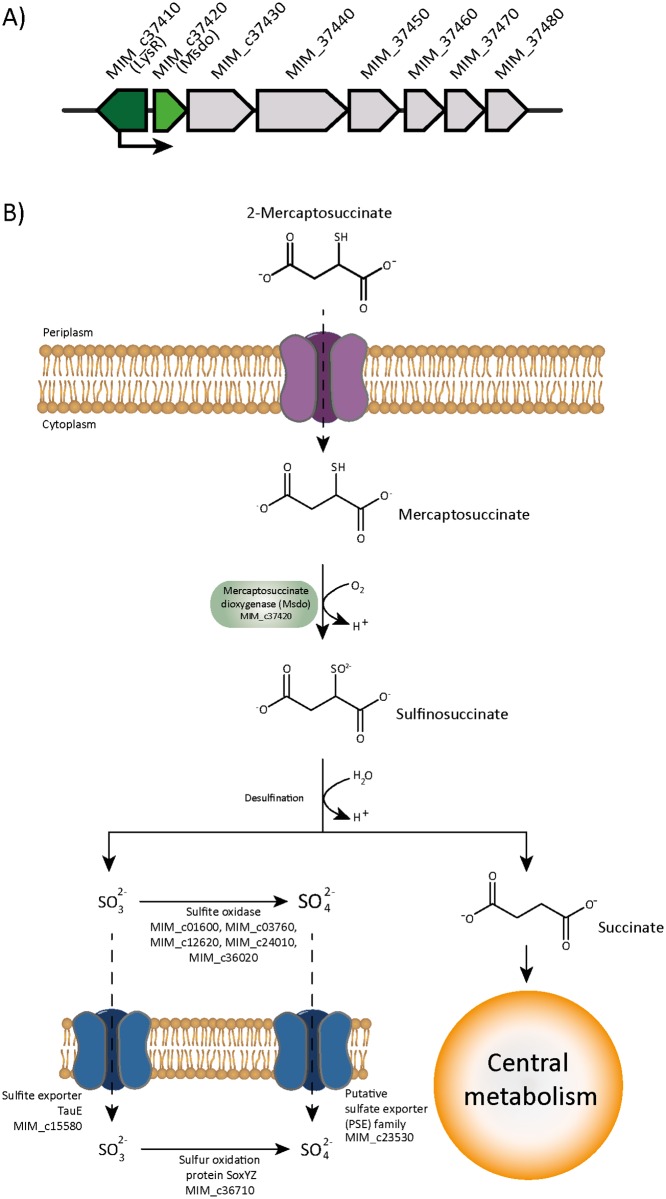
A) Gene neighborhood of transcriptional regulator LysR (MIM_c37410, dark green). Adjacent located genes: MIM_c37420, mercaptosuccinic acid dioxygenase (Msdo, light green); MIM_c37430, cystathionine beta lyase; MIM_c37440, rhodanese-related sulfurtransferase, MIM_c37450- MIM_c37480 amino acid ABC transporter PAAT family. B) Catabolism of mercaptosuccinate in *A*. *mimigardefordensis* strain DPN7^T^.

In addition, we observed that deletion of an upstream located *lysR* regulator with the locus tag MIM_c37410 also suppressed growth of the strain when MS was used as sole source of carbon and is, therefore, most probably the regulatory element of MIM_c37420 (Figs 8 and 9A) and the adjacent genes. Based on these data the degradation pathway of MS in *A*. *mimigardefordensis* strain DPN7^T^ can be proposed (Fig 9B). This pathway is similar to that of *V*. *paradoxus* strain B4 and differs only in the detoxification of the released sulfite as one of the final degradation products as discussed in the section below.

The other cupin 2 domain-containing protein (MIM_c14530), which attracted attention during proteome analysis, was considerably smaller and exhibited only about 21% amino acid similarity to that of Msdo_B4._ Moreover, the adjacent genes differ from the previously mentioned genes found in the neighborhood of Msdo_B4_. Furthermore, this protein showed also an increased expression when the cells were cultivated with the other organic sulfur compounds.

### Sulfite oxidation in *A*. *mimigardefordensis* strain DPN7^T^

Sulfite is released as one final degradation product in both investigated degradation pathways of DTDP and MS. In DTDP catabolism, sulfite is released during conversion of 3-sulfinopropionyl-CoA to propionyl-CoA by the Acd as described before [29]. Formation of sulfite during MS catabolism occurs via oxidation of MS by Msdo_DPN7_, yielding first the highly unstable sulfinosuccinate, which decomposes spontaneously to succinate plus sulfite [23]. This sulfite has to be detoxified because it is highly reactive and may alter coenzymes, cofactors or nucleic acids or might reduce protein disulfides to sulfonated cysteine derivatives, which could lead to restricted cell vitality [59,60]. In *V*. *paradoxus* strain B4, degradation of MS was well studied, and the fate of released sulfite was discussed by Brandt et al. [23]. Accordingly, sulfite is oxidized to sulfate by a molybdopterin oxidoreductase and the participation of a rhodanese domain-containing protein, which could be involved in cofactor synthesis needed for the molybdopterin oxidoreductase [23,24]. In *A*. *mimigardefordensis* strain DPN7^T^ the oxidation of sulfite is achieved differently [6].

The putative sulfur oxidation protein SoxYZ (MIM_c36710) has been identified during proteome analysis with a 7.6-fold increased protein level in cells cultivated with MS in comparison to cells cultivated with succinate. Additionally, the level of SoxYZ in cells cultivated with DTDP was 4.5-fold higher than in cells cultivated with succinate. An indirect involvement of this protein in MS and DTDP degradation e.g. by sulfite detoxification is likely. Based on genomic analysis, the emerging sulfite of DTDP degradation is probably either oxidized to sulfate by SoxYZ or via oxidation by one or several of the five sulfite dehydrogenases of the strain. The formed sulfate is subsequently exported via the putative sulfate exporter Pse (MIM_c23530) [3]. *Allochromatium vinosum* requires SoxYZ and the cytoplasmic sulfite-oxidizing enzymes for effective sulfite oxidation while the other components of the *sox* system are dispensable [61]. In *A*. *mimigardefordensis* strain DPN7^T^, it is possible that sulfite is exported to the periplasm via the sulfite exporter encoded by *tauE* (MIM_c15580), parallel to oxidation of sulfite in the cytoplasm to avoid its intracellular accumulation, as reported previously for *Cupriavidus necator* [62]. Subsequently, SoxYZ binds sulfite and further oxidation of this compound is achieved in the periplasm [63].

## Conclusion

In this study, we present for the first time a proteomic map of a member of the genus *Advenella*. Thereby, similarities in the proteomic pattern during degradation of the applied organic sulfur compounds were observed. The utilisation of the organic sulfur compounds provoked a stress response in cells of *A*. *mimigardefordensis* strain DPN7^T^ indicating an increased occurrence of misfolded proteins and therefore the increased synthesis of stress response proteins like DnaK, GroL, DegP or ClpB. Furthermore, the two regulatory elements of DTDP degradation (LysR and Xre) were determined and verified via phenotypical characterization of deletion mutants.

In addition, the key enzyme of MS degradation in this strain, Msdo_DPN7_ (MIM_c37420), was identified, and the reaction was confirmed by an enzyme assay. A *K*_*M*_ value of 0.2 mM for MS and a specific activity of 17.1 μmol mg^-1^ min^-1^ was determined. The gene encoding Msdo is regulated by a LysR transcriptional regulator. Single deletion mutants of this *lysR* (MIM_c37410) and *msdo* (MIM_c37420) resulted in restricted growth when MS was provided as the sole carbon source.

For an optimization of the metabolism of bacterial strains it is mandatory to understand the metabolic network of the precursor substances of interest. The display of proteomic maps during DTDP and MS utilization is a further important step towards successful metabolic engineering of *A*. *mimigardefordensis* strain DPN7^T^ aiming for higher PTE production.

## Supporting information

S1 Table Oligonucleotides. (PDF)

S2 Table Identified proteins with significantly high expression (ratio >2; mercaptosuccinate and 3,3´-dithiodipropionate in comparison to succinate). (PDF)

S3 Table Identified proteins with significantly high expression (ratio > 2; 3,3´-dithiodipropionate and 3-sulfinopropionate in comparison to propionate). (PDF)

S1 Fig Growth curves of *A*. *mimigardefordensis* strain DPN7^T^ cultures fed with propionate (blue, dimonds), 3,3´-dithiodipropionate (red, squares) and 3-sulfinopropionate (green, triangles). Each carbon source was provided at a concentration of 60 mM. Growth was monitored via a Klett Summerson photometer. The arrows indicate the sampling of each culture in the corresponding colour (blue, propionate; red, 3,3´-dithiodipropionate; green, 3-sulfinopropionate).
(TIF)

S2 Fig Growth curves of *A*. *mimigardefordensis* strain DPN7^T^ cultures fed with succinate (blue, dimonds), 3,3´-dithiodipropionate (red, squares) and 2-mercaptosuccinate (green, triangles). Each carbon source was provided at a concentration of 60 mM. Growth was monitored via a Klett Summerson photometer. The arrows indicate the sampling of each culture in the corresponding colour (blue, succinate; red, 3,3´-dithiodipropionate; green, 2-mercaptosuccinate).
(TIF)

S3 Fig Image of SDS polyacrylamide gelelectrophoresis of purification of Msdo_DPN7_. PageRuler^™^ Prestained Protein Ladder (Thermo Scientific, Schwerte, Germany) was used as Marker. Protein expression was accomplished in *E*. *coli* BL21 (DE3) pLysS pET-19b(+)::*msdo*_DPN7._ Displayed on the gel is the flow through of HisSpin-Trap^™^ column, the washing fraction and finally, the eluate.
(TIF)

S4 Fig Sequence alignment of amino acid sequences of Msdo from *A*. *mimigardefordensis* strain DPN7^T^ (MIM_c37420) compared to Msdo of *V*. *paradoxus* B4 (VPARA_1c41240). The alignment was generated using BioEdit software [64]. Cupin motifs 1 and 2 are accentuated; strictly conserved amino acid residues of the analysed sequences are highlighted in black.
(TIF)
