# Supplementary material for: Proteomic analysis of organic sulfur compound utilisation in Advenella mimigardefordensis strain DPN7T
Source: PLoS One. 2017 Mar 30;12(3):e0174256. doi: 10.1371/journal.pone.0174256 (PMC5373536; doi:10.1371/journal.pone.0174256)
Supplement: S1 Table — (PDF) [file pone.0174256.s001.pdf]

Supplemental Material

Table S1: Oligonucleotides

Primer	Sequence 5´-3´	Amplified fragment
U-Fw 14530 Del	AAAATCTAGAGATAACTGCGTTCATGCCAACC	Upstream flank Mim_c14530
U-Rv 14530 Del	AAAAGAATTCACGGCTTACTCCATAAAGTGAACG	
D-Fw 14530 Del	AAAAGAATTCGCCGGGTCTGTGACAGCAC	Downstream flank Mim_c14530
D-Rv 14530Del	AAAATCTAGACTCTTTTAAACGGCCGCCAG	
int-Fw_14530	GAAAGGTTTTGTCGATGATATCGAAG	Mim_c14530 internal gene fragment
int-RV 14530	GTCTTGCCATCAAAATGCTCTTC	
Ex-Fw 14530	CAGGTGTACGCAGGAAGACTCTAAG	Mim_c14530 external flank region
Ex-Rv 14530	CTTTACACCTAGTAATAATGCGCCATC	
U-Fw 37410 Del	AAAATCTAGAGATAGCGCAGCAAAGGAAACACG	Upstream flank Mim_c37410
U-Rv 37410 Del	AAAAGAATTCGATGCAGGGGCAGGTTACTGC	
D-Fw 37410 Del	AAAAGAATTCAGGGAATACCCTTAAAAACAAGCTCTTAAAAG	Downstream flank Mim_c37410
D-Rv 37410 Del	AAAATCTAGACAGGCCTTCCCAACGAGGC	
Int-Fw 37410	GAGCGCGCAGCATTTCATAG	Mim_c37410 internal gene fragment
Int-Rv 37410	GTCGGCCATCAGTCAACAAATG	
Ex_Fw 37410	GGGCGCCAGAAAAATCAGGAG	Mim_c37410 external flank region
Ex_Rv 37410	CTGCCTTTGATGATAGGCGTGTTG	
U-Fw 37420 Del	AAAATCTAGACGTCTCCCATACTCAGCCAATTG	Upstream flank Mim_c37420
U-Rv 37420 Del	AAAAGAATTCTTATTTCTCCTGTGATTTTTTCAAAGTATAGATAG	
D-Fw 37420 Del	AAAAGAATTCTGTCATCTGTCTCGCGAGCC	Downstream flank Mim_c37420
D-Rv 37420 Del	AAAATCTAGACTGCACAAAGCAGGATATCCCG	
Int-Fw 37420	CAGTGCCCAGCTGGATCGC	Mim_c37420 internal gene fragment
Int-Rv 37420	GACACCAACATCCATGATCTTGC	
Ex_Fw 37420	CCTTCCATTTGCTGCTGACCAAC	Mim_c37420 external flank region
Ex_Rv 37420	GCCAAAGTATTTGGTTGCTGCTACG	
U-Fw 37450 Del	AAAAGAATTCTATCTATTTGATGCTCCCCGCAAGAC	Upstream Flank Mim_c37450-37480
U-Rv 37450 Del	AAAAGAATTCCGTTCGCTCCGGTTGTGTTT	
D-Fw 37480 Del	AAAAGAATTCGTCCCTTTATTTGCGTGGTGTATACC	Downstream Flank Mim_c37450-37480
D-Rv 37480 Del	AAAATCTAGAGCCTGCTGGACAAGTTGATGC	

Int-Fw 37450	GCAGAAACCACAGCCCCC	Mim_c37450-37480 internal gene fragment
Int-Rv 37480	CACGTTCATGGGTAGCTTGAGAG	
Ex-Fw 37450	CTGGAAAAGGAGATTGGCGTGAC	Mim_c37450-37480 external gene fragment
Ex-Rv 37450	CACGTTCATGGGTAGCTTGAGAG	
U-Fw 31370	AAAAGGATCCCCGCCCATGCCCTGAATGAG	Upstream Flank MIM_c31370
U-Rv 31370	AAAAGAATTCACCAAGCTTGGCCCCCGC	
D-Fw 31370	AAAAGAATTCAGGTGCAGCGCGTTTTGAATG	Downstream Flank MIM_c31370
D-Rv 31370	AAAATCTAGACACCGCTTTGGCGTCAGTTTC	
Int-Fw 31370	GAGCTGGCGCTGGGGACC	MIM_c31370 internal gene fragment
Int-Rv 31370	CGATGTGCCTTGAAGCACCG	
Ex-Fw 31370	CATCTGCTTGCTATCGTGCGC	MIM_c31370 external flank region
Ex-Rv 31370	CAATTGCAATATGACATTGACCTGTTAGG	
U-Fw 31360	AAAATCTAGACGAATTGCTTGATCGCCACTCC	Upstream Flank MIM_c31360
U-Rv 31360	AAAAGAATTCCGCGCCAGGACGGCC	
D-Fw 31360	AAAAGAATTCGGTGGCCATTTCATTGAATGCTG	Downstream Flank MIM_c31360
D-Rv 31360	AAAATCTAGACAGGCCTGGGCCAGATCCAG	
Int-Fw 31370	GATACGCTGCTGACGTTTTCATTC	MIM_c31360 internal gene fragment
Int-Rv 31370	CATGCGGAATTACGCGAGATCG	
Ex-Fw 31370	CGCTGGAGCTGGCGCTG	MIM_c31360 external flank region
Ex-Rv 31370	GCAGGTGAGCCACCTCTTCG	
pET19_37420_fwd	AAACTCGAGATGACTTCAAAGCAAGACAAGCAGGTTGCC	Expression of Msdo (MIM_c37420)
pET19_37420_rev	AAAGGATCCTTAGCCCTTGCTCCGCTGGGTTTTG	

Restriction sites are underlined
