# Supplementary material for: Proteomic analysis of organic sulfur compound utilisation in Advenella mimigardefordensis strain DPN7T
Source: PLoS One. 2017 Mar 30;12(3):e0174256. doi: 10.1371/journal.pone.0174256 (PMC5373536; doi:10.1371/journal.pone.0174256)
Supplement: S2 Table — (PDF) [file pone.0174256.s002.pdf]

Table S2: Identified proteins with significantly high expression (ratio >2 mercaptosuccinic acid and 3,3´-dithiodipropionic acid in comparison to succinic acid).

Spot number	Protein description	Gene	Locus tag MIM_cXXXXX	MS/ Suc	DTDP / Suc	MS/ DTDP
80	TRAP transporter solute receptor, DctP family		01930	8.2	2.4	3.5
129	DnaK suppressor protein-like	*dksA*	05080	2.6	1.2	2.2
50	*S*-Adenosylmethionine synthase	*metK*	05180	1.1	4.9	0.2
36	Carboxy-terminal-processing protease		07410	1.7	2.9	0.6
131	ATP synthase subunit delta	*atpH*	07490	4.1	5.2	0.8
139	Hypothetical protein		07750	1.7	2.5	0.7
59	Outer membrane porin protein		08090	4.0	3.3	1.2
60	Outer membrane porin protein		08090	6.7	1.5	4.4
100	Glutamine-binding periplasmic protein	*glnH1*	09310	1.8	0.7	2.6
155	Glutamine-binding periplasmic protein	*glnH1*	09310	1.8	3.3	n.d.
99	Molybdopterin binding domain-containing protein		10340	2.9	2.2	1.3
159	Chaperone protein DnaK	*dnaK*	11560	0.7	3.1	0.2
150	Putative Bug-like extracytoplasmic solute-binding receptor, TTT family		11970	7.3	8.2	0.9
107	Putative Sua5/YciO/YrdC/YwlC family protein		12270	2.5	2.3	1.1
110	Putative Sua5/YciO/YrdC/YwlC family protein		12270	2.0	3.4	0.6
82	Dihydrodipicolinate synthase		12280	0.5	2.4	0.2
15	Ferritin-like domain-containing protein		12960	3.9	3.5	1.1
16	Ferritin-like domain-containing protein		12960	2.9	4.0	0.7
29	Ferritin-like domain-containing protein		12960	4.8	3.9	1.2
10	Malate dehydrogenase	*mdh2*	13210	0.8	2.4	0.3
116	Malate dehydrogenase	*mdh2*	13210	0.6	3.4	0.2
93	Succinate dehydrogenase iron-sulfur subunit	*sdhB*	13260	2.4	4.8	0.5
94	Succinate dehydrogenase iron-sulfur subunit	*sdhB*	13260	2.7	7.3	0.4
151	Putative Bug-like extracytoplasmic solute-binding receptor, TTT family		13470	4.1	8.6	0.5
109	Acetoacetyl-CoA reductase	*phaB*	13540	5.1	4.4	1.2
125	Alkylhydroperoxidase AhpD core domain-containing protein		14470	2.7	1.4	1.9
128	Cupin 2 domain-containing protein		14530	14.7	1.7	8.6
20	30S Ribosomal protein S6	*rpsF*	14830	0.7	2.0	0.4
5	50S Ribosomal protein L9	*rplI*	14860	1.7	2.4	0.7
12	Putrescine-binding periplasmic protein		15080	0.8	1.8	0.5
106	3-Hydroxyacyl-CoA dehydrogenase		15790	1.2	1.7	0.7
17	Superoxide dismutase [Fe]	*sodB*	15840	1.4	2.3	0.6
114	Superoxide dismutase [Fe]	*sodB*	15840	1.4	1.9	0.7
96	Electron transfer flavoprotein subunit beta	*etfB*	16510	2.0	2.4	0.8
132	Electron transfer flavoprotein subunit alpha		16520	1.0	0.8	n.d.
104	Putative protease, peptidase family M48		16530	2.2	1.2	1.9
157	Putative protease, peptidase family M48		16530	4.0	3.8	1.1

Table S2 continued

Spot number	Protein description	Gene	Locus tag MIM_cXXXXX	MS/ Suc	DTDP / Suc	MS/ DTDP
45	Fumarylacetoacetase	*fah*	16690	3.3	2.0	1.7
121	Aspartate-semialdehyde dehydrogenase	*asd*	16730	2.7	2.2	1.2
73	Putative extracellular solute-binding protein		16750	1.9	1.0	1.9
117	Putative argininosuccinate lyase		16970	6.4	1.9	3.5
126	Hypothetical protein		18130	3.7	4.4	0.7
71	Succinate-CoA ligase subunit alpha	*sucD*	18290	0.3	1.6	0.2
1	Thioredoxin	*trxA*	18410	0.3	1.9	0.1
119	Thioredoxin	*trxA*	18410	3.4	1.4	2.4
8	Putative Fe-S cluster assembly scaffold protein IscU	*nifU*	18540	1.3	7.9	0.2
137	Fe-S Protein assembly chaperone HscA	*hscA*	18570	6.1	6.2	n.d.
138	Fe-S Protein assembly chaperone HscA	*hscA*	18570	3.3	4.5	0.8
139	Fe-S Protein assembly chaperone HscA	*hscA*	18570	1.7	2.5	0.7
53	*Sn*-glycerol-3-phosphate-binding periplasmic protein UgpB	*ugpB*	19070	2.3	2.3	1.0
58	*Sn*-glycerol-3-phosphate-binding periplasmic protein UgpB	*ugpB*	19070	4.8	1.3	3.7
30	Putative outer membrane protein assembly factor YaeT	*bamA*	19410	2.6	3.4	0.8
93	Putative Bug-like extracytoplasmic solute-binding receptor, TTT family		19700	2.4	4.8	0.5
123	Putative enoyl-CoA hydratase		20690	3.2	3.2	1.0
125	Nitrogen regulatory protein P-II	*glnB1*	21350	2.7	1.4	1.9
124	Nitrogen regulatory protein P-II	*glnB1*	21350	2.2	1.7	1.3
40	Inosine-5'-monophosphate dehydrogenase	*guaB*	21640	3.2	1.7	1.9
127	Outer membrane protein A	*ompA*	22870	2.7	6.3	n.d.
33	Putative tail-specific protease		22960	7.7	8.9	0.9
136	Putative tail-specific protease		22960	5.8	5.9	1.0
64	Phospho-2-dehydro-3-deoxyheptonate aldolase	*aroG*	23210	2.4	4.7	0.5
66	Phospho-2-dehydro-3-deoxyheptonate aldolase	*aroG*	23210	2.1	3.8	0.6
38	Do-like serine protease	*degP*	23840	4.0	6.1	0.7
40	Do-like serine protease	*degP*	23840	3.2	1.7	1.9
42	Do-like serine protease	*degP*	23840	7.1	10.2	0.7
43	Do-like serine protease	*degP*	23840	5.8	1.5	3.8
46	Do-like serine protease	*degP*	23840	0.8	2.4	0.3
53	Do-like serine protease	*degP*	23840	2.3	2.3	1.0
141	Do-like serine protease	*degP*	23840	10.3	1.4	7.3
142	Do-like serine protease	*degP*	23840	8.5	1.0	8.9
144	Do-like serine protease	*degP*	23840	5.7	3.0	1.9
145	Do-like serine protease	*degP*	23840	5.8	4.3	1.4
130	Alkyl hydroperoxide reductase subunit C	*ahpC*	24360	2.2	3.4	0.7
132	Alkyl hydroperoxide reductase subunit C	*ahpC*	24360	1.0	0.8	n.d.
72	Phosphoserine phosphatase	*serB*	24410	2.6	1.0	2.4
60	Putative CBS domain-containing nucleotidyltransferase		24430	6.7	1.6	4.4

Table S2 continued

Spot number	Protein description	Gene	Locus tag MIM_cXXXXX	MS/ Suc	DTDP / Suc	MS/ DTDP
47	Outer membrane porin protein		24600	1.9	2.9	0.7
48	Outer membrane porin protein		24600	4.1	2.0	2.0
49	Outer membrane porin protein		24600	2.7	0.7	4.0
52	Outer membrane porin protein		24600	1.7	1.2	1.4
54	Outer membrane porin protein		24600	2.3	1.0	2.3
55	Outer membrane porin protein		24600	3.1	1.0	3.0
56	Outer membrane porin protein		24600	1.6	0.5	3.2
57	Outer membrane porin protein		24600	2.0	0.8	2.6
58	Outer membrane porin protein		24600	4.8	1.3	3.7
60	Outer membrane porin protein		24600	6.7	1.5	4.4
72	Putative glutathione S-transferase		24890	2.6	1.1	2.4
35	Urease subunit alpha	*ureC2*	27040	0.6	2.3	0.3
68	Glyceraldehyde-3-phosphate dehydrogenase	*gap*	27790	1.4	2.3	0.6
122	Putative protein ElaB		27900	3.2	1.5	2.1
113	Biotin synthase	*bioB*	28390	1.9	3.9	0.5
25	Putative beta-lactamase hydrolase-like protein		28620	0.7	5.6	0.1
61	L-Asparaginase	*ansA2*	28910	3.4	1.6	2.2
46	Serine hydroxymethyltransferase	*glyA*	29220	0.8	2.4	0.3
73	Putative TRAP-type transporter, periplasmic component		29310	1.9	1.0	1.9
95	Putative phospholipase/carboxylesterase/thioesterase		29340	2.0	6.2	0.3
84	Putative oxidoreductase		29910	1.0	2.6	0.4
86	Putative oxidoreductase		29910	1.0	2.5	0.4
63	Protein TolB	*tolB*	29980	0.9	2.4	0.4
23	Putative peptidoglycan-associated lipoprotein		29990	0.2	1.8	0.1
25	Putative peptidoglycan-associated lipoprotein		29990	0.7	5.6	0.1
11	Methylisocitrate lyase	*prpB*	31320	1.4	3.3	0.4
27	Methylisocitrate lyase	*prpB*	31320	1.9	4.5	0.4
88	Methylisocitrate lyase	*prpB*	31320	1.8	11.0	0.2
115	Methylisocitrate lyase	*prpB*	31320	1.8	5.1	0.4
14	3-Mercaptopropionate dioxygenase	*mdo*	31400	1.6	9.8	0.2
148	Putative Bug-like extracytoplasmic solute-binding receptor, TTT family		31430	2.5	1.9	1.4
24	Putative LysM domain-containing BON superfamily protein		33130	1.1	3.1	0.4
128	Putative LysM domain-containing BON superfamily protein		33130	14.7	1.7	8.6
52	Putative carboxypeptidase G2		36310	1.7	1.2	1.4
91	Putative sulphur oxidation protein SoxYZ	*soxYZ*	36710	7.5	4.5	1.7
133	Putative monothiol glutaredoxin, Grx4 family		37030	1.4	4.2	0.3
154	Putative Bug-like extracytoplasmic solute-binding receptor, TTT family		37240	2.4	2.1	1.1
79	Curved DNA-binding protein	*cbpA*	37340	1.7	0.8	2.0
153	Cupin 2 domain-containing protein		37420	10.9	1.4	n.d.

Table S2 continued

Spot number	Protein description	Gene	Locus tag MIM_cXXXXX	MS/ Suc	DTDP / Suc	MS/ DTDP
83	Putative periplasmic amino acid-binding protein		37450	22.7	3.1	7.4
85	Putative periplasmic amino acid-binding protein		37450	5.6	8.6	0.7
101	Amino acid ABC transporter ATP-binding protein		37480	6.8	0.9	7.5
102	Amino acid ABC transporter ATP-binding protein		37480	7.0	3.1	2.3
105	Amino acid ABC transporter ATP-binding protein		37480	1.0	2.4	0.4
155	Amino acid ABC transporter ATP-binding protein		37480	1.8	3.3	n.d.
65	Putative polysaccharide export protein Wza		37830	2.7	3.4	0.8
67	Putative polysaccharide export protein Wza		37830	1.7	1.9	0.9
25	Putative extracellular solute-binding protein, family 1		37890	0.7	5.6	0.1
118	Type VI secretion system protein, Hcp1-like family		38060	1.7	1.0	1.8
93	Putative Bug-like extracytoplasmic solute-binding receptor TctC		39280	2.4	4.8	0.5
72	Putative TRAP transporter solute receptor, DctP family		39430	2.6	1.1	2.4
2	Branched-chain amino acid ABC transporter substrate-binding protein		39890	1.6	1.8	0.9
3	Branched-chain amino acid ABC transporter substrate-binding protein		39890	2.1	0.5	4.2
4	Branched-chain amino acid ABC transporter substrate-binding protein		39890	3.7	2.3	1.6
