# Supplementary material for: Proteomic analysis of organic sulfur compound utilisation in Advenella mimigardefordensis strain DPN7T
Source: PLoS One. 2017 Mar 30;12(3):e0174256. doi: 10.1371/journal.pone.0174256 (PMC5373536; doi:10.1371/journal.pone.0174256)
Supplement: S3 Table — (PDF) [file pone.0174256.s003.pdf]

Table S3: Identified proteins with significantly high expression (ratio > 2 3,3´-dithiodipropionic acid and 3-sulfinopropionic acid in comparison to propionate).

Spot number	Protein description	Gene	Locus tag MIM_cXXXXX	DTDP/ P	3SP/ P	DTDP/ 3SP
69	NAD(P)-binding Rossmann-fold domain-containing protein	*yhfK*	01310	1.1	2.9	0.3
28	TRAP transporter solute receptor, DctP family	*dctP*	01930	1.2	3.0	0.5
29	TRAP transporter solute receptor, DctP family	*dctP*	01930	1.3	3.9	0.4
31	TRAP transporter solute receptor, DctP family	*dctP*	01930	1.6	2.3	0.7
32	TRAP transporter solute receptor, DctP family	*dctP*	01930	2.2	4.6	0.5
93	Hypothetical protein		02120	1.5	0.5	3.2
144	Putative hybrid peroxiredoxin	*hyPrx5*	03810	2.9	2.2	n.d.
64	Lysine-arginine-ornithine-binding periplasmic protein	*argT*	03840	1.6	0.5	3.3
131	S-Adenosylmethionine synthase	*metK*	05180	2.9	2.1	n.d.
79	Putative periplasmic Fe2+ transport protein		06180	2.4	4.2	0.6
92	Putative lipoprotein		06490	2.0	2.1	0.9
54	Putative Bug-like extracytoplasmic solute-binding receptor, TTT family	*bug*	08730	1.3	2.5	0.6
49	Glutamine-binding periplasmic protein	*glnH1*	09310	6.7	1.3	5.1
149	Glutamine-binding periplasmic protein	*glnH1*	09310	2.2	2.3	n.d.
68	Glutamine transport ATP-binding protein GlnQ	*glnQ2*	09330	2.1	1.4	1.5
59	Putative phasin		10360	1.9	0.6	3.1
61	Putative phasin		10360	6.4	1.3	4.7
89	Ferric uptake regulation protein	*fur1*	11490	1.4	2.0	0.7
91	Ferric uptake regulation protein	*fur1*	11490	1.5	2.3	0.6
3	Chaperone protein DnaK	*dnaK*	11560	9.2	4.0	2.4
4	Chaperone protein DnaK	*dnaK*	11560	6.4	3.4	1.9
127	Chaperone protein DnaK	*dnaK*	11560	4.2	2.5	n.d.
185	Isocitrate dehydrogenase [NADP]	*icd1*	11690	2.3	4.0	n.d.
37	Putative Bug-like extracytoplasmic solute-binding receptor, TTT family	*bug*	11920	2.5	2.9	0.8
25	Glutamate/aspartate periplasmic-binding protein	*gltI*	12140	1.5	2.6	0.7
29	Glutamate/aspartate periplasmic-binding protein	*gltI*	12140	1.3	3.9	0.4
34	Glutamate/aspartate periplasmic-binding protein	*gltI*	12140	2.6	3.4	0.8
121	Putative lipoprotein		12290	2.0	3.2	n.d.
143	Putative ectoine/hydroxyectoine ABC transporter solute-binding protein EhuB	-	12320	2.8	1.8	n.d.
160	Peptidyl-prolyl cis-trans isomerase	-	12560	3.7	2.0	n.d.
161	Peptidyl-prolyl cis-trans isomerase	-	12560	2.8	2.0	n.d.
171	Peptidyl-prolyl cis-trans isomerase	-	12560	2.4	1.8	n.d.
51	Ferritin-like domain-containing protein		12960	1.0	2.0	0.5
53	Ferritin-like domain-containing protein		12960	1.9	3.1	0.6
152	Ferritin-like domain-containing protein		12960	5.3	3.2	n.d.
187	Malate dehydrogenase	*mdh2*	13210	2.4	3.1	n.d.

Table S3 continued

Spot number	Protein Description	Gene	Locus tag MIM_cXXXXX	DTDP/ P	3SP/ P	DTDP/ 3SP
72	Cupin 2 domain-containing protein		14530	0.4	0.2	2.8
81	Cupin 2 domain-containing protein		14530	4.0	4.4	0.8
94	Cupin 2 domain-containing protein		14530	2.4	0.5	6.8
158	Cupin 2 domain-containing protein		14530	2.3	2.3	n.d.
78	Cupin 2 domain-containing protein		14530	5.6	4.3	1.2
76	Cupin 2 domain-containing protein		14530	3.6	1.4	2.6
52	3-Hydroxyacyl-CoA dehydrogenase		15790	2.6	2.1	1.3
56	3-Hydroxyacyl-CoA dehydrogenase		15790	2.3	1.0	2.1
61	Adenylate kinase	*adk*	15800	6.4	1.3	4.7
60	Superoxide dismutase [Fe]	*sodB*	15840	3.0	1.9	1.5
62	Superoxide dismutase [Fe]	*sodB*	15840	3.0	2.1	1.4
167	Putative peroxiredoxin-like protein		16370	3.6	1.3	n.d.
41	Putative peroxiredoxin-like protein		16370	0.8	4.5	0.2
180	Electron transfer flavoprotein subunit alpha	*etfA*	16520	3.0	3.4	n.d.
153	Inorganic pyrophosphatase	*ppa*	18360	2.8	1.7	n.d.
116	Leucine-, isoleucine-, valine-, threonine-, and alanine-binding protein	*braC2*	19030	2.0	2.1	0.8
192	Leucine-, isoleucine-, valine-, threonine-, and alanine-binding protein	*braC2*	19030	1.7	3.0	n.d.
118	Elongation factor Ts	*tsf*	19340	2.8	1.6	1.8
195	Elongation factor Ts	*tsf*	19340	3.3	3.9	n.d.
41	Putative inner membrane transport protein		19690	0.8	4.5	0.2
93	Organic hydroperoxide resistance protein	*ohr1*	21060	1.5	0.5	3.2
174	Nitrogen regulatory protein P-II	*glnB1*	21350	3.1	2.2	n.d.
65	Putative transamidase		21680	1.8	0.5	5.4
130	Trigger factor	*tig*	22030	4.2	2.1	n.d.
2	Chaperone protein ClpB	*clpB*	22270	5.2	7.4	0.7
93	Hypothetical protein		23110	1.5	0.5	3.2
35	Putative Bug-like extracytoplasmic solute-binding receptor, TTT family	*bug*	23770	1.4	4.2	0.3
25	Putative Bug-like extracytoplasmic solute-binding receptor, TTT family	*bug*	23770	1.5	2.6	0.6
9	Serine protease Do	*degP*	23840	8.5	7.5	1.1
108	Serine protease Do	*degP*	23840	4.2	12.0	0.3
184	Serine protease Do	*degP*	23840	2.3	0.1	n.d.
18	Putative parvulin-type peptidyl-prolyl cis-trans isomerase		24270	1.5	0.7	2.2
142	Putative parvulin-type peptidyl-prolyl cis-trans isomerase		24270	2.9	1.1	n.d.
158	AhpC: alkyl hydroperoxide reductase subunit C (EC 1.11.1.15)	*ahpC*	24360	2.3	1.3	n.d.
160	AhpC: alkyl hydroperoxide reductase subunit C (EC 1.11.1.15)	*ahpC*	24360	3.7	2.0	n.d.
11	Outer membrane porin protein		24600	2.5	5.6	0.4
137	Outer membrane porin protein		24600	1.5	3.0	n.d.
96	Lactoylglutathione lyase	*gloA*	26650	1.1	4.1	0.2

Table S3 continued

Spot number	Protein description	Gene	Locus tag MIM_cXXXXX	DTDP/ P	3SP/ P	DTDP/ 3SP
143	Phosphoribosylaminoimidazole-succinocarboxamide synthase	*purC*	27710	2.8	1.8	n.d.
152	Molybdopterin adenylyltransferase	*mog*	27740	5.3	3.2	n.d.
179	Phosphoglycerate kinase	*pgk*	27780	2.7	5.3	n.d.
21	Glyceraldehyde-3-phosphate dehydrogenase	*gap*	27790	2.6	3.7	0.7
147	putative lysine-arginine-ornithine-binding periplasmic protein		28380	2.4	1.9	n.d.
42	Putative oxidoreductase		29910	1.2	0.4	2.8
59	Putative oxidoreductase		29910	1.9	0.6	3.1
64	Putative L-amino acid-binding periplasmic protein		30070	1.6	0.5	3.3
33	TRAP transporter solute receptor, DctP family		30680	1.4	3.1	0.5
114	Methylisocitrate lyase	*prpB*	31320	4.4	1.2	3.7
2	Fe/S-dependent 2-methylisocitrate dehydratase	*acnD*	31340	5.2	7.4	0.5
13	Acyl-CoA dehydrogenase	*acd*	31390	3.5	5.0	0.7
14	Acyl-CoA dehydrogenase	*acd*	31390	17.3	21.0	0.9
16	Acyl-CoA dehydrogenase	*acd*	31390	4.0	6.3	0.6
17	Acyl-CoA dehydrogenase	*acd*	31390	7.1	9.4	0.7
117	Acyl-CoA dehydrogenase	*acd*	31390	2.7	4.9	0.6
122	Acyl-CoA dehydrogenase	*acd*	31390	2.3	2.9	n.d.
138	Acyl-CoA dehydrogenase	*acd*	31390	9.7	12.7	n.d.
194	Acyl-CoA dehydrogenase	*acd*	31390	1.5	3.3	n.d.
179	Acyl-CoA dehydrogenase	*acd*	31390	2.7	5.3	n.d.
49	3-Mercaptopropionate dioxygenase	*mdo*	31400	6.7	1.3	5.1
152	3-Mercaptopropionate dioxygenase	*mdo*	31400	5.3	3.2	n.d.
141	Putative Bug-like extracytoplasmic solute-binding receptor TctC, TTT family		31420	3.5	2.2	n.d.
36	Putative Bug-like extracytoplasmic solute-binding receptor TctC, TTT family		31430	3.3	3.1	0.9
1	60 kDa chaperonin GroEL	*groL*	32020	1.6	3.3	0.5
128	60 kDa chaperonin GroEL	*groL*	32020	2.4	2.4	n.d.
129	60 kDa chaperonin GroEL	*groL*	32020	2.5	1.4	n.d.
5	60 kDa chaperonin GroEL	*groL*	32020	3.9	3.0	1.3
47	50S ribosomal protein L25	*rplY*	32420	2.0	0.8	2.6
90	Putative LysM domain-containing BON superfamily protein		33130	4.7	6.4	0.6
171	Putative LysM domain-containing BON superfamily protein		33130	2.4	1.8	n.d.
126	Putative outer membrane protein, OmpA family		36930	2.6	2.0	n.d.
8	Methylmalonate semialdehyde dehydrogenase [acylating]	*iolA*	37360	0.6	2.2	0.3
191	UDP-N-acetyl-D-mannosamine dehydrogenase	*wecC*	37790	2.3	4.7	n.d.
75	Single-stranded DNA-binding protein	*ssb2*	38600	2.6	5.2	0.5
144	Putative Bug-like extracytoplasmic solute-binding receptor TctC, TTT family		39280	2.9	2.2	n.d.
41	Putative Bug-like extracytoplasmic solute-binding receptor TctC, TTT family		39280	0.8	4.5	0.2
24	Putative TRAP transporter solute receptor, DctP family		39430	1.5	2.3	0.7

Table S3 continued

Spot number	Protein description	Gene	Locus tag MIM_cXXXXX	DTDP/ P	3SP/ P	DTDP/ 3SP
24	Putative TRAP transporter solute receptor, DctP family	-	39430	1.5	2.3	0.7
122	Branched-chain amino acid ABC transporter substrate-binding protein	-	39890	2.3	2.9	n.d.
123	Branched-chain amino acid ABC transporter substrate-binding protein	-	39890	2.2	2.9	n.d.
171	50S ribosomal protein L7/L12	*rplL*	40640	2.4	1.8	n.d.
115	Elongation factor Tu	*tuf1*	40710	4.4	8.7	0.5
115	Elongation factor Tu	*tuf1*	40710	4.4	8.7	0.5
188	Elongation factor Tu	*tuf1*	40710	3.7	5.7	n.d.
189	Elongation factor Tu	*tuf1*	40710	1.6	2.0	n.d.
190	Elongation factor Tu	*tuf1*	40710	6.1	5.3	n.d.
64	Succinyl-CoA:3-ketoacid-coenzyme A transferase subunit A	*lpsI*	23580	1.6	0.5	3.3
