# Supplementary figures and images for: Proteomic analysis of organic sulfur compound utilisation in Advenella mimigardefordensis strain DPN7T
Source: PLoS One. 2017 Mar 30;12(3):e0174256. doi: 10.1371/journal.pone.0174256 (PMC5373536; doi:10.1371/journal.pone.0174256)

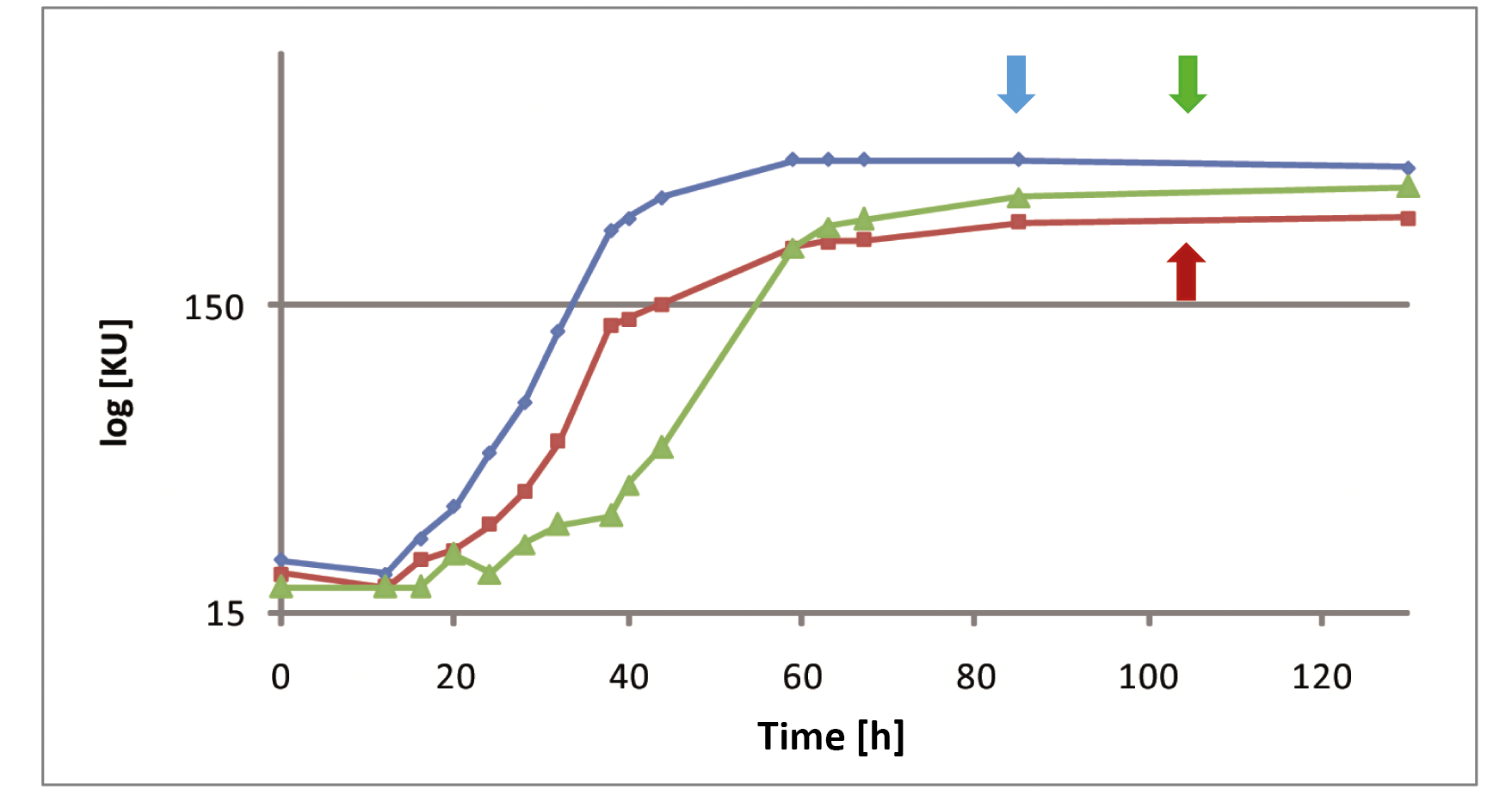

Supplement: S1 Fig — Each carbon source was provided at a concentration of 60 mM. Growth was monitored via a Klett Summerson photometer. The arrows indicate the sampling of each culture in the corresponding colour (blue, propionate; red, 3,3´-dithiodipropionate; green, 3-sulfinopropionate). (TIF) [file pone.0174256.s004.tif]

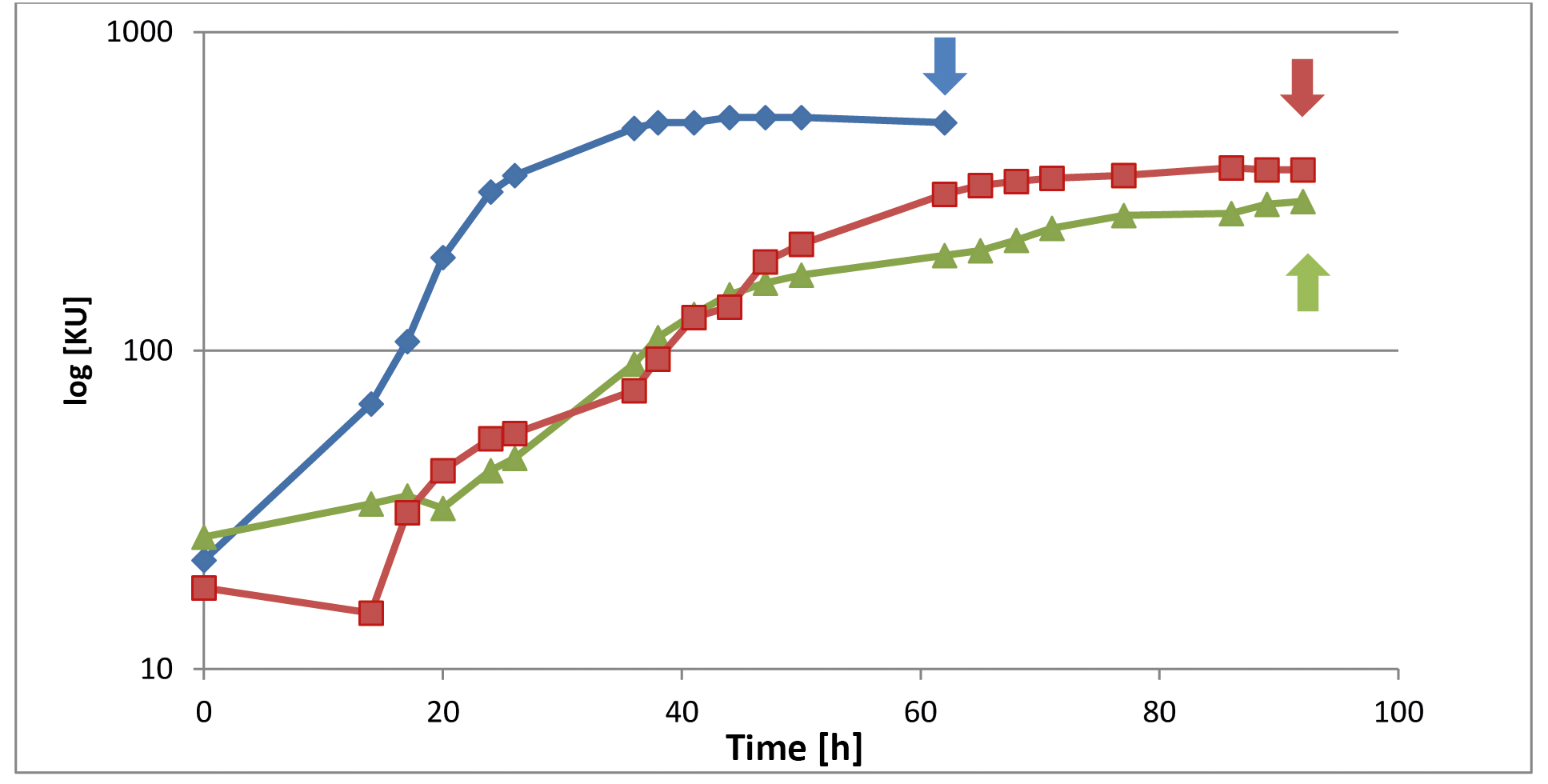

Supplement: S2 Fig — Each carbon source was provided at a concentration of 60 mM. Growth was monitored via a Klett Summerson photometer. The arrows indicate the sampling of each culture in the corresponding colour (blue, succinate; red, 3,3´-dithiodipropionate; green, 2-mercaptosuccinate). (TIF) [file pone.0174256.s005.tif]

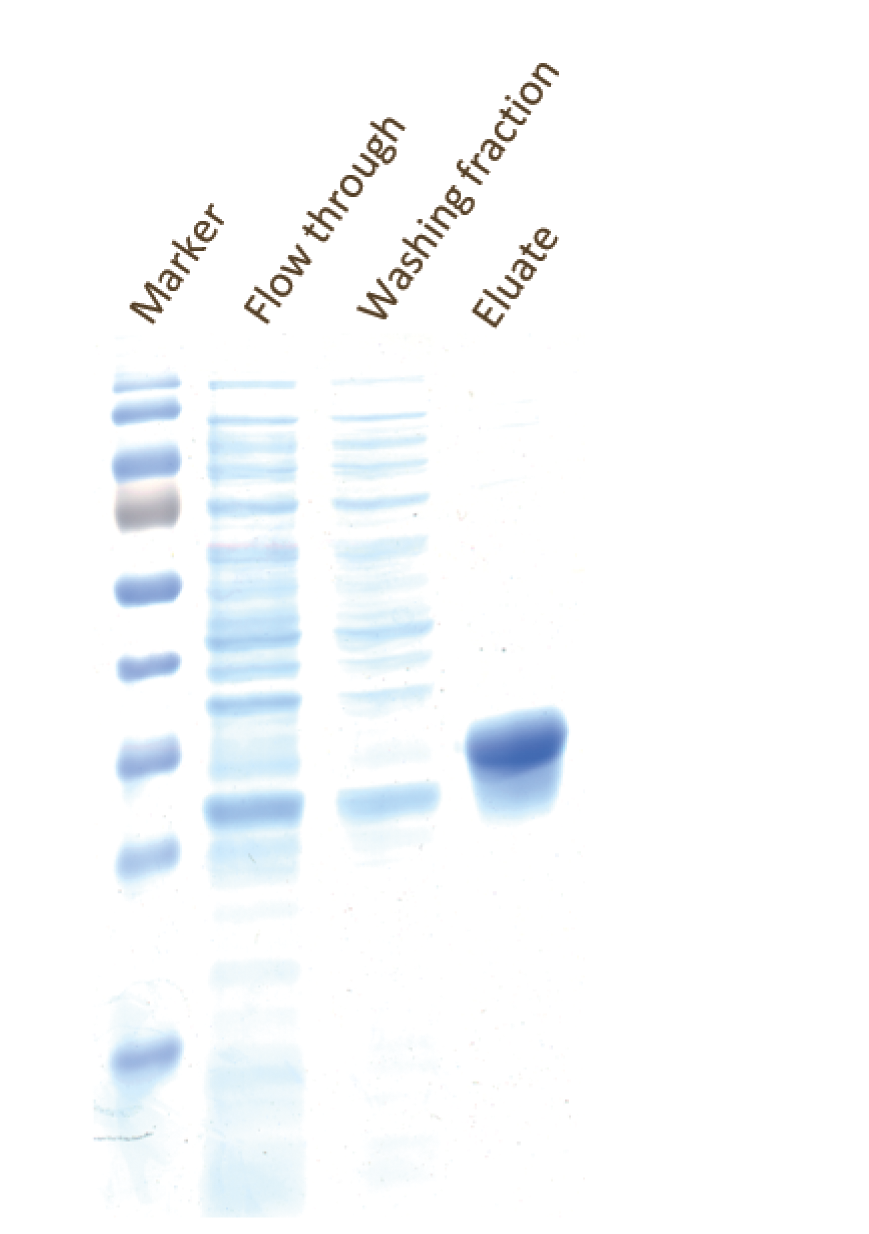

Supplement: S3 Fig — PageRuler™ Prestained Protein Ladder (Thermo Scientific, Schwerte, Germany) was used as Marker. Protein expression was accomplished in E. coli BL21 (DE3) pLysS pET-19b(+)::msdoDPN7. Displayed on the gel is the flow through of HisSpin-Trap™ column, the washing fraction and finally, the eluate. (TIF) [file pone.0174256.s006.tif]

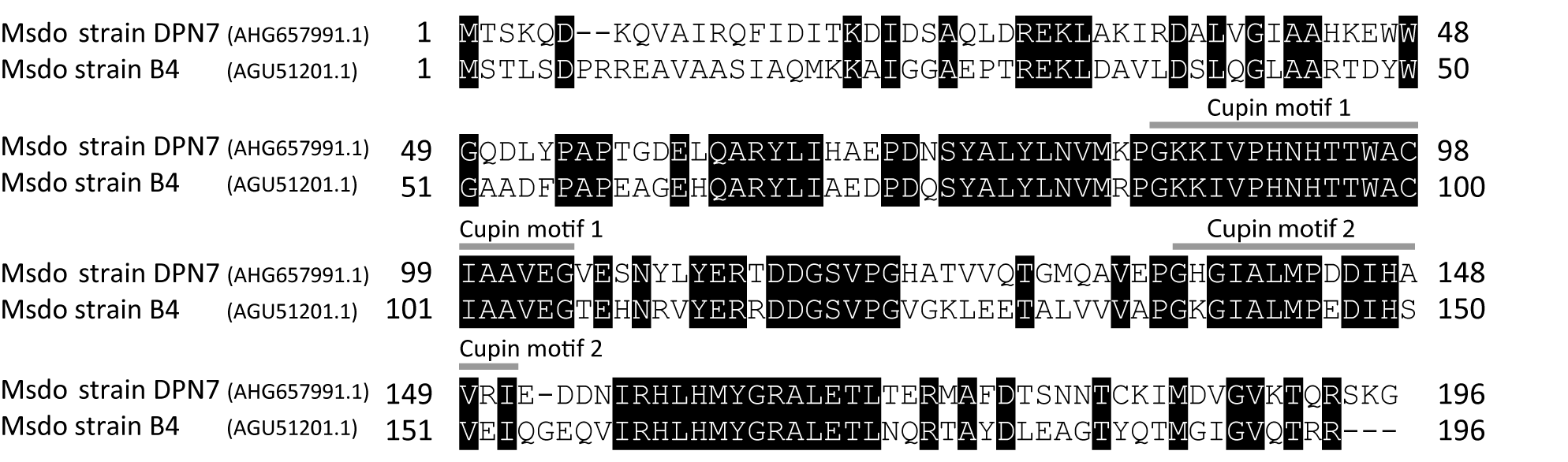

Supplement: S4 Fig — The alignment was generated using BioEdit software [64]. Cupin motifs 1 and 2 are accentuated; strictly conserved amino acid residues of the analysed sequences are highlighted in black. (TIF) [file pone.0174256.s007.tif]
